# Distinct small non-coding RNA landscape in the axons and released extracellular vesicles of developing primary cortical neurons and the axoplasm of adult nerves

**DOI:** 10.1080/15476286.2021.2000792

**Published:** 2021-12-09

**Authors:** Raquel Mesquita-Ribeiro, Rafael Sebastián Fort, Alex Rathbone, Joaquina Farias, Cristiano Lucci, Victoria James, Jose Sotelo-Silveira, Maria Ana Duhagon, Federico Dajas-Bailador

**Affiliations:** a School of Life Sciences, University of Nottingham, Nottingham, UK; b Laboratorio de Interacciones Moleculares, Facultad de Ciencias, Universidad de la República, Montevideo, Uruguay; c Departamento de Genómica, Instituto de Investigaciones Biológicas Clemente Estable, Montevideo, Uruguay; d Polo de Desarrollo Universitario “Espacio de Biología Vegetal del Noreste”, Centro Universitario Regional Noreste, UdelaR, Uruguay; e School of Veterinary Medicine and Science, University of Nottingham, Nottingham, UK

**Keywords:** Neurons, axon, extracellular vesicles, sncRNAs, miRNAs, tRNA-derived fragments

## Abstract

Neurons have highlighted the needs for decentralized gene expression and specific RNA function in somato-dendritic and axonal compartments, as well as in intercellular communication via extracellular vesicles (EVs). Despite advances in miRNA biology, the identity and regulatory capacity of other small non-coding RNAs (sncRNAs) in neuronal models and local subdomains has been largely unexplored.

We identified a highly complex and differentially localized content of sncRNAs in axons and EVs during early neuronal development of cortical primary neurons and in adult axons *in* *vivo*. This content goes far beyond miRNAs and includes most known sncRNAs and precisely processed fragments from tRNAs, sno/snRNAs, Y RNAs and vtRNAs. Although miRNAs are the major sncRNA biotype in whole-cell samples, their relative abundance is significantly decreased in axons and neuronal EVs, where specific tRNA fragments (tRFs and tRHs/tiRNAs) mainly derived from tRNAs Gly-GCC, Val-CAC and Val-AAC predominate. Notably, although 5ʹ-tRHs compose the great majority of tRNA-derived fragments observed *in* *vitro*, a shift to 3ʹ-tRNAs is observed in mature axons *in* *vivo*.

The existence of these complex sncRNA populations that are specific to distinct neuronal subdomains and selectively incorporated into EVs, equip neurons with key molecular tools for spatiotemporal functional control and cell-to-cell communication.

## Introduction

Neural circuit development and function relies on the establishment of a diverse and well-defined set of connections between the different areas of the nervous system, which requires neurons to develop an exceptionally complex cellular structure defined by large intracellular distances and highly compartmentalized specializations of cellular functions. In this context, the specific localization and translation of RNAs allows the spatial and temporal control of protein expression required for neurons to function and respond to the various environmental cues [1,2]. Although the widespread acceptance of mRNA axonal transport and translation did not happen overnight [3–5], the specific regulation of local protein synthesis is now fully recognized as an important functional characteristic of highly polarized and morphologically complex neurons [6–8].

Following the early discovery of core axonal translational machinery, last decade’s combined progress in both axon isolation methods and next-generation sequencing (RNA-seq) has led to the unravelling of the mRNA complexity in the axonal territory [9–11]. The importance of this localization and its role in neuronal function and development has been further enhanced by the observation that the identity of these mRNAs can vary between neuronal subtypes, axonal subdomain and developmental time [2,12]. In this regard, although the majority of axonal transcriptome datasets have been obtained using cultured primary neurons, the importance of this mechanism has also been confirmed *in* *vivo* [11,13,14] further establishing the capacity for fine tuning of RNA distributions and localized protein expression in adult tissues [13,15]. The dynamic nature of these regulatory mechanisms was demonstrated by the rapid changes in the translatome during conversion from growth cone to synaptic terminals [16], and during the establishment of neuronal wiring *in vivo*, where a subset of axonally translated mRNAs encodes for functionally linked proteins matching temporal axonal needs [17]. In effect, the evidence for axonal protein synthesis has expanded from a solely developmental stage to a demonstrated mechanism in most neuronal processes, including neuron specification, survival, plasticity, injury response and regeneration [18–21]. Axonal translation has thus emerged not just as a mechanism to dynamically control protein content in the axon compartment, but also as a retrograde communication process that expands the potential for axon to soma signal integration [19,22–24].

This decentralization of gene expression towards sub-cellular domains has recently added a novel and exciting dimension, with the role of extracellular vesicles in the transcellular transport of mRNAs and non-coding RNAs [25–27], which has opened an entirely new perspective on cellular communication in the brain [28]. It suggests that functional compartmentalization in the nervous system is not only dependent on the architecture of single neurons, but also on the local spatial neighbourhoods comprised by multiple neuronal cohorts [8]. Intriguingly, despite the demonstration that neuronal cultures can regulate EV release [29–31], and the relatively abundant literature about glia-neuron mechanisms [28,32,33], the profile and role of non-coding RNAs in inter-neuronal communication and the development of neuronal networks remains less explored.

As part of the efforts to understand the role of local translation in neurons, the full mRNA landscape in localized neuronal domains has been increasingly refined [1,12,34,35]. However, although a relatively high extent of the genome is transcribed, only a small proportion is made of mRNA [36], a ratio that points towards the existence of an important amount of non-coding RNA-dependent regulatory processes [37]. Indeed, the number of functions ascribed to non-coding RNAs (ncRNAs) has grown to incorporate most biological processes, including roles in axon development, neuron connectivity and regeneration [38–42]. In the axon, although the long non-coding and circular RNAs have attracted growing attention [43], most of the research efforts have centred on miRNAs [39,44]. Indeed, early studies in peripheral neurons demonstrated distributed localization of miRNAs in cellular compartments [45,46], with later reports also describing soma-restricted miRNAs capable of regulating axon pathfinding by mediating global changes in gene expression [47,48]. In addition to this cell body function, miRNA machinery proteins and miRNAs have been found in the axons of both central and peripheral nervous system neurons [49–53] and more recently in the synaptic compartment [54]. Attempts to characterize the population of axonal miRNAs started by combining microarray expression profiling with RT-PCR using primary cultures of superior cervical ganglion neurons [55] and cortical neurons [53,56], and was accompanied by the description of their capacity to locally regulate axonal development and function [24,39,44,46,50,57–59].

When extending beyond miRNAs, the reports on the role of other short non-coding RNAs and their specific localization in neuronal compartments has been rather limited. Despite this, there has been a growing awareness of the increasing complexity of small RNAs derived from longer non-coding RNA sequences and the role they can play in gene expression at pre- and post- transcriptional level. Among them, a new class of ‘non-micro-short’ RNAs that map to known tRNA genes has been uncovered [60]. Depending on the site of cleavage, the tRNA-derived small RNAs (tsRNAs) can be divided into two main types, tRFs (approx. 14–30 nt) and derived from mature or precursor tRNAs, and tRNA halves, known as tRHs or tiRNAs (29–50 nt), which are produced instead by specific cleavage at the mature tRNA anticodon loop [61]. Unlike miRNAs and siRNAs, which depend on Dicer or type III RNase enzymatic cleavage, tRHs/tiRNAs are cleaved by angiogenin. Functionally, this class of sncRNAs can bind to multiple RNA binding proteins and have been proposed as novel regulators of translation at different levels, depending on cell status or subtype [60,62]. Interestingly, both tRFs and tRHs have been linked to the occurrence and development of cancer [61], while their association with AGO has led to a proposed role in RNA silencing [63]. The reported link to cancer mechanisms offers a tantalizing glimpse at their potential involvement in axonal development, where cell growth and invasive cell dynamics are also required cell mechanisms [64].

Along with the realization that virtually all RNA classes can give rise to a well-defined and reproducible repertoire of smaller fragments, the spectrum of known sncRNAs has continued to expand in recent years, including those derived from small nuclear RNAs (snRNAs) and small nucleolar RNAs (snoRNAs) [65,66]. Their biogenesis and accumulation appear to be dependent on cell type, developmental stage and physiological conditions, but despite recent advances, and more available knowledge of sncRNA content in peripheral neuron systems, small RNA-seq datasets from sub-cellular domains in CNS neurons are still scarce and centred around miRNAs [44,54]. Here, we have used mouse primary cortical neuron cultures grown in compartmentalized microfluidic chambers and the axoplasm of mature motor and sensory axons to isolate and sequence those small RNAs present in whole-neurons, extracellular vesicles and axons during early neuronal development *in vitro* and in adult axons. We demonstrate the existence of a complex and differentially localized content of sncRNAs that goes far beyond miRNAs and includes most known sncRNAs and derived fragments, from tRNAs, snoRNAs, snRNAs, Y RNAs, vault RNAs (vtRNAs) and others. These findings will help to unravel the intricate sncRNA landscapes in distinct sub-cellular neuronal domains *in vitro* and *in vivo*, providing evidence of mechanistic importance and potentiating the investigation of their functional roles in neuronal communication processes in the nervous system.

## Results

### RNA isolation from sub-cellular and extracellular domains

**Figure 1.**
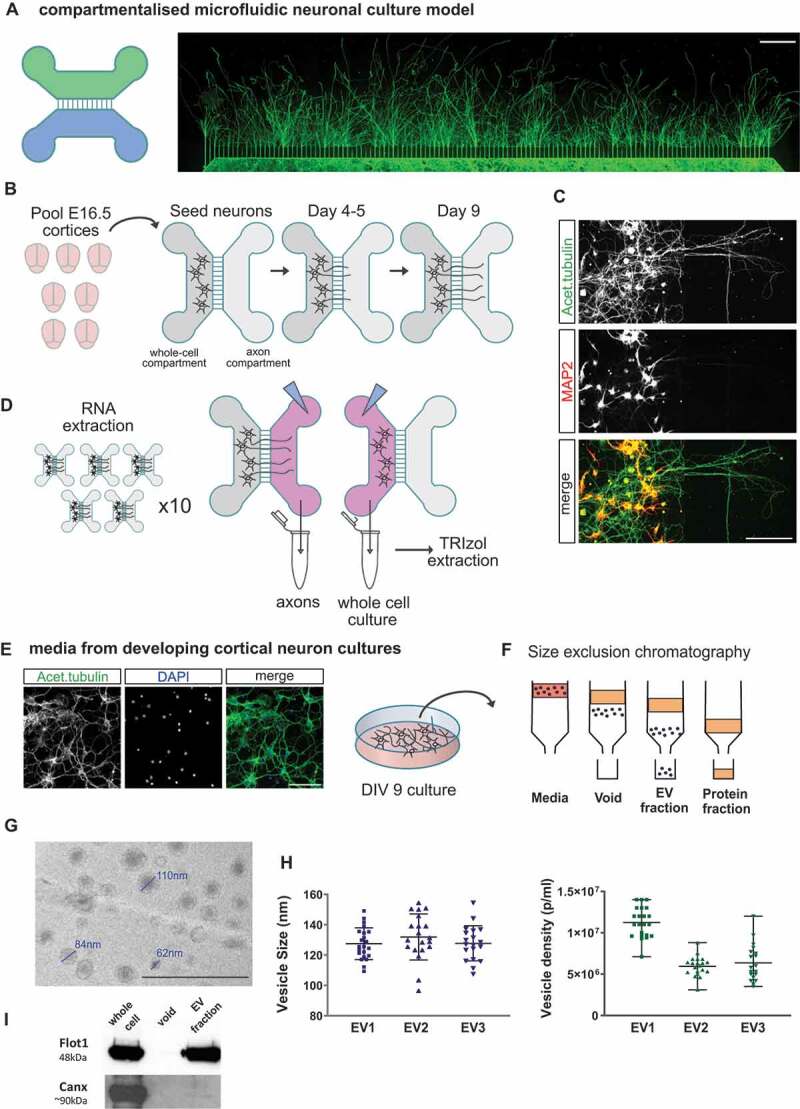
Primary cortical neuron culture models for RNA isolation from neuronal compartments: whole cell, axon and neuron-derived extracellular vesicles. (A) Schematic representation of a compartmentalized microfluidic chamber and immunofluorescence image of cortical primary neurons grown in this device. In this model two culture channels are connected by 150um microgrooves allowing compartmentalization of axons from their neuron cell bodies and dendrites. Acetylated tubulin staining (green) shows the presence of axons across the full axonal compartment at day 9 in culture (scale bar 500 µm). (B) Diagrammatic representation of the experimental preparation protocol using compartmentalized cultures. Post-mitotic cortical neurons are prepared from a E16.5 litter (total of ~7-9 cortices) and seeded onto the designated WC (whole cell) channel. As the culture develops, axons extend across the microgrooves and into the axonal channel at ~4-5 days in culture. WC and pure axonal fractions are harvested for RNA extraction at day 9 in culture to allow for extensive axon coverage in the axon channel. (C) Immunofluorescence image of cortical neurons in a microfluidic chamber and labelled with the dendritic marker MAP2 (red), which indicates how dendrites do not extend to the designated axonal (AX) side of the device at this stage in culture. On the other hand, the axon-enriched marker acetylated tubulin (green) is present in both WC and AX channels (scale bar 150 µm). (D) The WC and AX fractions of 40–50 microfluidic devices were collected and pooled for each biological replicate. To collect axonal pure fractions, TRIzol reagent was applied to the axonal channel whilst maintaining hydrostatic pressure in the WC channel with PBS, thus preventing contamination from the WC side. WC was collected immediately thereafter. (E) Neuron-derived extracellular vesicles (EV) were obtained from media collected from day 9 of standard primary cortical cultures as depicted (green: acetylated tubulin; blue: dapi; scale bar 100 µm). (F) Diagrammatic representation of the size exclusion chromatography method for the isolation of the EV fraction from neuronal culture media, highlighting how EVs are separated from the media’s protein fraction. (G) EV fractions were visualized by transmission electron microscope revealing the expected vesicular structure (scale bar 500 nm). (H) Nanoparticle tracking analysis (NTA) showing average particle size and mean particle density measured for the three EV preparations used for RNA extraction and small RNA-sequencing. Data expressed as mean ± range of NTA measurements. (I) Western blotting of the isolated EV fraction showing the presence of EV marker flotilin-1 (Flot1) and near absence of calnexin (Canx), an endoplasmatic reticulum marker largely absent in small EVs (< 200 nm).

To investigate the profile of sncRNAs in different compartments relevant to neuronal function and communication, we first cultured mouse primary cortical neurons in microfluidic chambers, which allow the isolation of axons from their cell bodies (Fig. 1A). A combined pool of ~7-9 brains from E16.5 embryos were used for each neuron seeding onto the designated somatodendritic (whole-cell) compartment of microfluidic chambers. Axons were allowed to grow through the microgrooves over time, covering the respective axonal side compartment area (Fig. 1B). After 9 days in culture, only cortical axons are seen in the axon compartment of the chamber, as demonstrated by the lack of staining for the dendritic marker MAP2, which confirms the inability of dendrites to reach beyond the separating microgrooves over the course of the experiment (Fig. 1C). Using this culture system, we could independently extract RNA samples corresponding to the mainly somatodendritic compartment, defined throughout this study as whole-cell samples (WC), or the exclusively axonal (AX) domain of primary cortical neurons. The total RNA of WC or AX fractions isolated from approximately 50 chambers were pooled together (Fig. 1D), and this process was repeated three times from different neuronal preparations to obtain the final number of independent samples for sequencing (n = 3).

Due to the small volumes used to culture cortical neurons in compartmentalized microfluidic chambers, the isolation of extracellular vesicles (EVs) required instead the use of culture media from primary neurons grown at similar cell densities and developmental stage but cultured in 6-well plates (Fig. 1E). Individual EV samples were obtained from independent neuronal preparations as previously done for microfluidic chambers. Each EV RNA sample was extracted from the EV fraction isolated after size-exclusion chromatography (Fig. 1F), used to obtain an EV enriched sample, depleted of non-EV components, particularly soluble proteins and protein complexes present in the culture media [67–70]. Importantly, size-exclusion chromatography avoids the use of high-pressure forces during ultracentrifugation steps, thus conserving the structural integrity and biological activity of the vesicles [71,72]. Characterization of the EV fraction by transmission electron microscopy and nanoparticle tracking analysis confirmed the presence of vesicular structures with a cup-shape morphology typical of EV preparations (Fig. 1G), and with a size distribution based at around 130 nm (Fig. 1H). These parameters, and the observed vesicle density in all samples (Fig. 1H), are in agreement with previous studies on EVs [29,30,73,74]. In addition, the EV characterization was further confirmed by the presence of flotillin-1, a well-known protein marker for EVs (Fig. 1I), and the absence from the EV fraction of calnexin (Canx), a protein associated with the endoplasmic reticulum and unlikely to be present in small (< 200 nm) EVs (Fig. 1I; [75]).

### Global sncRNA sequencing data analysis

To characterize the repertoire of sncRNAs in the different neuronal and extracellular compartments, we sequenced the libraries of small RNAs from our samples and the resultant sequencing reads were mapped to the genome. The total raw read counts in all samples show comparable numbers, with the percentage of mapped reads per sample averaging 83.1%, an indicator of good overall sequencing accuracy (Table S1). Pearson inter-sample correlation analysis for raw read counts showed high correlation coefficients among independent repeats for each of the samples (WC, AX and EV), with those of EVs and WC being the most distant and EVs and AX the more similar, when comparing the different sample types (Fig S1A). Principal component analysis (PCA) performed on sncRNA mapping of total reads also showed clear clustering by sample type (Fig S1B), with the first three components of the analysis representing most of the observed variance (Suppl Fig S1B inset). Although axonal samples are the most variable (average correlation of 0.73), the combined Pearson analysis among sample replicates (0.9 average) and PCA analysis demonstrates the high intra-sample similarity and reproducibility in biological replicates from each of the neuronal compartments (WC and AX) and EVs.

Despite our initial global analysis showing comparable levels of raw read counts between samples (Fig S1C), these are not adequate to compare expression levels among different sample types, as they can be affected by transcript length, total number and sequencing biases [76]. The normalization methods commonly performed, such as reads per kilobase and transcripts per million (i.e. RPKM, FPKM and TPM), are based on total or effective counts, and perform less well with samples that have heterogeneous distributions with highly and differentially expressed features, which can skew count distribution [77]. To overcome this, we performed UpperQuartile (UQ) edgeR normalization implemented with edgeR (Fig S1D, Fig S2A; Table S2), which disregards highly variable and/or highly expressed features [77], and which allowed the subsequent direct comparison of WC, AX and EVs samples.

Another important aspect in the investigation of RNA levels in axons, EVs and any other low input samples *in vitro* is the potential problem of RNA contaminations in culture media, with recent studies reporting miRNAs present in foetal bovine serum (FBS) [78]. The fact that our cortical primary neurons are grown in serum-free media should prevent exogenous contamination from FBS, as reported by Wei et al. [79]. However, a more recent study by Auber and co-authors [80] identified how serum-free media supplements, such as B27, can carry miRNAs that co-purify with EVs. Among the current efforts to remove this confounding factor, key quality control approaches use the assessment of so-called bellwethers or contamination-betraying candidates [81], of which the main ones are miR-122-5p and miR-451a [80]. As such, our sncRNA-seq showed negligible number of reads for these miRNAs in the EV fractions (miR-122-5p: 1,1,0 reads and miR-451a: 30, 2, 5 in each independent sample), which provides support for the lack of significant exogenous contamination. It must be noted that unlike the majority of studies reporting the potential contamination from RNAs in the culture media, we do not use ultracentrifugation for EV isolation, relying instead on column fractionation. It is thus possible that this method, together with serum-free media, provides a more efficient experimental approach for the avoidance of contaminant RNAs in EV fractions.

### Relative composition of read size distribution and RNA biotypes

**Figure 2.**
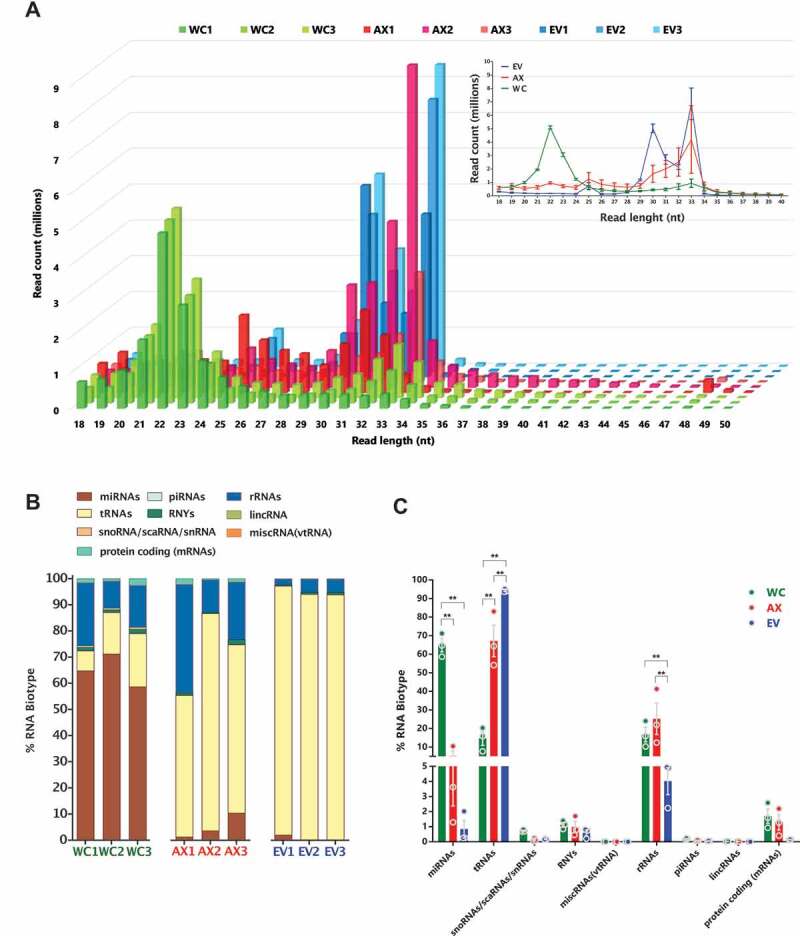
Read size distribution and relative RNA biotype composition in the three neuronal compartments. (A) Read length (nt) distribution plot for the individual samples (main plot) and average read length distribution of the biological replicates for each compartment (mean ± s.e.m., upper right plot) showing the higher abundance of 22nt long reads in the WC, whereas the AX and EV samples presented their highest peaks at 33nt and 30nt. (B) Percentage distribution of total reads for the RNA biotypes assigned in the analysis, shown as per independent sample. (C) Mean percentage distribution of total reads for each RNA biotype in the three neuronal compartments investigated (mean ± s.e.m). Comparisons between neuronal compartments demonstrate that miRNAs represent a far greater proportion of WC reads in relation to AX and, particularly EV samples, whereas reads mapping to tRNAs compose a higher proportion of AX and EV reads. Two-way ANOVA with Tukey’s multiple comparison post-hoc test, * p-value < 0.05; ** p-value < 0.01. Whole Cell (WC), Axon (AX) and Extracellular Vesicles (EV).

**Figure 3.**
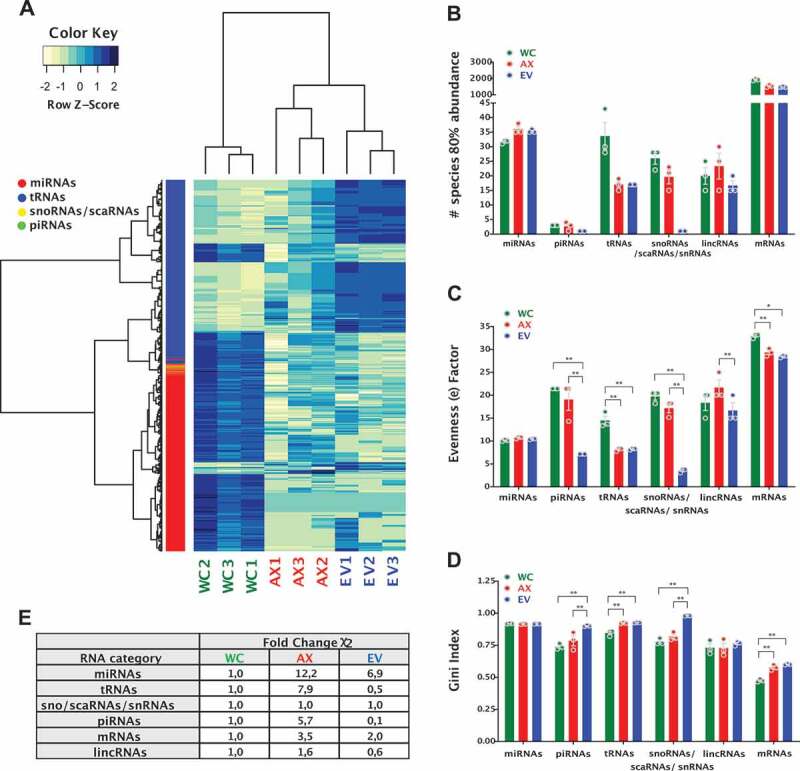
Differentially expressed non-coding RNA genes and analysis of inequality/heterogeneity of the RNA repertoire. (A) Heatmap of differentially expressed genes; 379 DEGs (miRNAs, tRNAs, snoRNAs/scaRNAs and piRNAs), FDR ≤ 0.01 and absolute log2(FC) ≥ 1) for the different samples, with expression shown as Z-score of log2 normalized counts (two-way hierarchical clustering distance measured by Euclidean and Ward clustering algorithms). (B) Bar plot for the number of specific RNAs that make 80% of the total normalized read counts for each of the RNA biotypes in the different samples (mean ± s.e.m.). Bar plot of the (C) Evenness factors and (D) Gini coefficients, reflecting the inequality of abundance distribution of the indicated RNA biotypes in the neuronal and EV compartments. Higher evenness factors or lower Gini coefficients correspond to lower inequality. (E) Analysis of heterogeneity of the sncRNA repertoire between samples. For each RNA biotype, a sum of squared errors (χ2 value) was calculated among samples, after normalization. The χ2 value of EV and AX samples was compared to the WC samples. Fold change of χ2 values higher than 1 reflects the increased heterogeneity. Two-way ANOVA with Tukey’s multiple comparison post-hoc test, * p-value < 0.05; ** p-value < 0.01. Whole Cell (WC), Axon (AX) and Extracellular Vesicles (EV).

As a first step towards the investigation of the sncRNA landscape in sub-cellular domains and EVs, we analysed the length distribution of the mapped reads in the different samples. In Fig. 2A, we show how WC samples have a higher amount of 21–23 nt reads compared to AX and EVs, with the latter two showing a significantly higher amount of 30–33 nt reads, a first indication of the different distribution of sncRNAs in neuronal sub- and extra- cellular domains. Interestingly, although the overall neuronal profile that stems from merging WC, AX and EV data sets would show a bimodal distribution, this pattern is highly dependent on the neuronal sub-domain analysed. In particular, the size distribution of EVs is skewed towards longer reads, a pattern that shares clear similarities with AX samples. Identity assignment of the observed peaks to RNA biotypes denotes that those reads centred at 22 nt, and mostly present in WC samples, are largely composed by miRNAs (Fig. 2B). On the other hand, the 30–33 nt peak that is enriched in AX and EV samples is highly abundant of tRNA derived small RNAs (tsRNAs). All the annotated RNA species were categorized into 7 classes, with the vast majority of mapped reads corresponding to fragments of smaller RNAs rather than full-length transcripts, with the exception of miRNA and piRNA reads. The percentage distribution of reads that mapped to specific RNA biotypes showed that WC sncRNAs are composed by nearly 70% of miRNA reads, but this decreases to less than 10% in AX and EVs (Fig. 2C). In the case of tsRNAs, their distribution is largely concentrated in AX and EV samples, with a striking 70–90% of sncRNA reads corresponding to them in these samples. Accordingly, the cumulative expression distribution plots of miRNAs, tRNAs and sRNAS (sno/scaRNAs and snRNAs) reflects the differences in abundance of these features among the compartments, which is not seen for those fragments corresponding to protein coding genes (Fig S2B). Although rRNA fragments are significantly less abundant in EVs compared to WC and AX, in the case of fragments of sno/scaRNAs, snRNAs, RNYs and vtRNAs the initial analysis of percentage RNA biotype distribution is largely similar in all samples. However, a more in-depth analysis showing differences in their specific fragment identity is shown in subsequent sections. At this first stage of global analysis, the characteristics of the sncRNA landscape found in each compartment were also investigated by differential expression, shown as a Z-score of normalized counts, and based on the two-way hierarchical clustering distance measured by Euclidean and Ward clustering algorithms. As shown in Fig. 3A, and consistent with our previous analysis of percentage distribution of RNA biotypes (miRNAs, tRNAs, sno/scaRNAs and piRNAs), miRNAs are significantly enriched in WC samples corresponding largely to cell bodies, while levels of tsRNAs are comparatively higher in both axons and EVs.

### Inequality and heterogeneity of sncRNA biotypes

The next step in the analysis of the sncRNA repertoire in subcellular and extracellular domains was to investigate their overall diversity and distribution of representation, exploring their inequality and heterogeneity. In results consistent with previous reports in glioma stem cells [78], we show that just about 30 miRNAs make 80% of the miRNA abundance in WC, AX and EV compartments (Fig. 3B). However, although small RNAs mapped to tRNAs are the most abundant sncRNA type in AX and EV samples, reads mapped to just 15 parental tRNAs make up to 80% of their expression in these compartments, which is half the number observed in cell bodies. This provided an early indication of the specific sncRNA expression, or inequality, observed in the samples. For a more objective representation of this phenomenon, we followed a two-step approach to study evenness and inequality, as recently detailed by Krichevsky and co-authors [78]. First, we compared the similarity of the population size for each RNA type in all samples by calculating the evenness factor (e), which defines that e % of RNA species can account for (100-e) % of total abundance (Fig. 3C). Originally described for population studies, low evenness equates to a large disparity within the sample, while a high evenness factor is associated with a more equal representation. As a second approach, we calculated the Gini coefficient/index, which is a measure of statistical dispersion that was initially developed to represent inequality in economics’ population studies (Fig. 3D). In brief, a Gini index <0.2 represents high equality while >0.5 denotes severe disparity. Interestingly, although the population of miRNAs show no difference in their equality of representation among samples, we observed a significant increase in the inequality of EV content when analysing tsRNAs, piRNAs, sno/scaRNAs and snRNA fragments. In the case of tsRNAs, both the AX and EV samples showed increased inequality compared to WC (Fig. 3B-D).

Further to the inequality analysis, we extended our approach to the investigation of the heterogeneity of the RNA repertoire by normalizing the reads to the total read number within an individual RNA category, and then addressing the sum of squared errors (χ^2^ value), which measures the amount of variability in the data. As such, higher χ^2^ value reflects higher diversity/heterogeneity in RNA composition (Fig. 3E). Overall, this analysis further reinforced the growing concept that RNA biotypes are processed, transported and distributed using highly specific and/or localized mechanisms that generate differential expression patterns. For example, miRNAs show similar inequality in subcellular and extracellular compartments, but the miRNA population in both AX and EVs have higher levels of heterogeneity compared to the whole cell. Thus, although similar number of miRNAs account for the majority of miRNA reads across the three compartments, the specific miRNAs expressed in AX and EVs are more diverse than those found in the WC. This is not just a reflection of the relative abundance of an RNA species in each compartment, since tsRNAs, which are the most abundant RNA biotype in both AX and EV samples, show dramatically different heterogeneity levels. As shown in Fig. 3E, EVs have a more unequal but less heterogeneous sample compared to WC, while AX tsRNAs have comparable levels of inequality to EVs but accompanied by a higher level of heterogeneity.

Overall, the analysis of distribution and inequality provides strong evidence for the selective and specific compartmentalization of sncRNAs. To address their potential role in cellular processes we decided to investigate their detailed biotype variability and localization commonalities, e.g. the specific identity of those regularly present among the most abundant species. For this, our subsequent experimental analysis focused on the separate profiling of the most relevant sncRNA biotypes, to explore and contemplate their specific characteristics and distribution.

### miRNAs

The profiling of miRNAs in different neuronal compartments from multiple species has demonstrated the existence of a rich and complex repertoire [44]. However, the appearance of a clear axon miRNA signature has been difficult to discern, with discrepancies probably due to specific differences in experimental models, methodologies, and the lack of significant number of data sets currently available. As recently highlighted by Corradi and Baudet [44], a higher overlap seems to be found when similar profiling methods are used, with RNA sequencing showing the greatest reliability in this regard, independent of experimental model. Here, we provide the first direct comparison of miRNAs in WC, AX and EV sncRNA sequencing datasets, at a crucial stage in the development of axon and neuron connectivity in primary cortical neurons.

**Figure 4.**
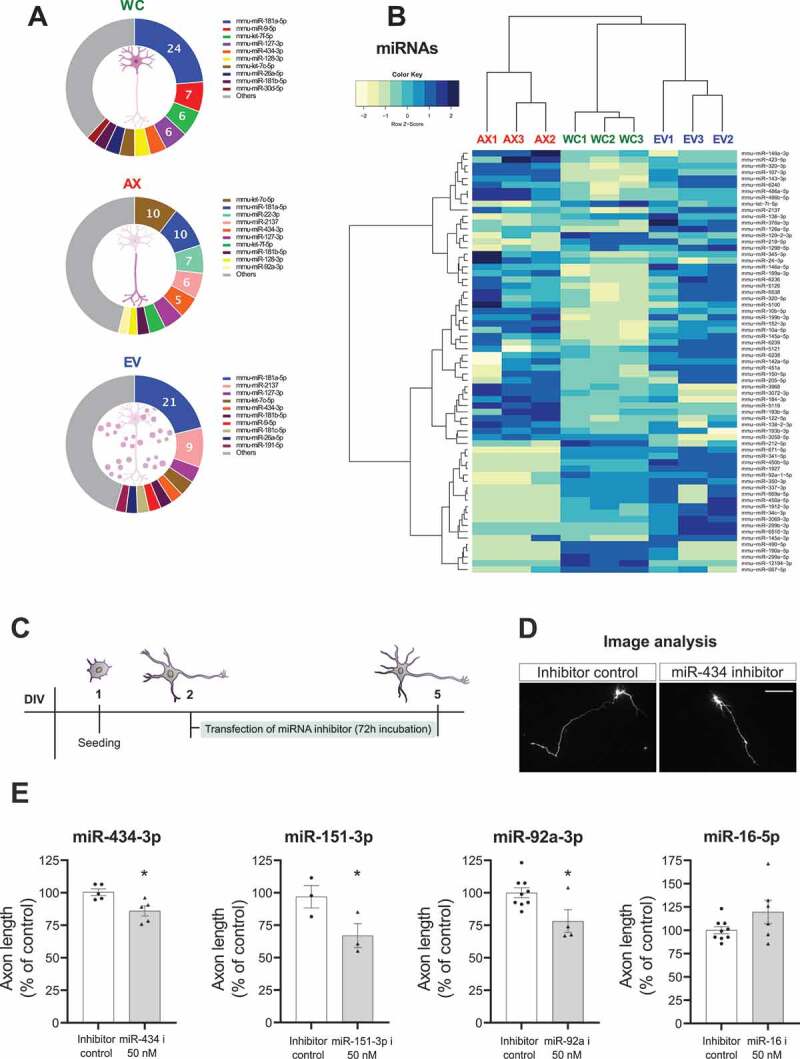
Characterization of miRNAs in neuronal subcellular and extracellular compartments and selective assessment of axonal growth effects. (A) Relative read abundance of miRNAs in WC, AX and EVs showing the most expressed miRNAs in each neuronal and EV compartment (percentage of the average normalized miRNA counts for each compartment). (C) Heatmap of the differential expression of miRNAs (67 DEGs, FDR ≤ 0.01 and absolute log2(FC) ≥ 1) for the different samples, shown as Z-score of log2 normalized counts (two-way hierarchical clustering distance measured by Euclidean and Ward clustering algorithms). Whole Cell (WC), Axon (AX) and Extracellular Vesicles (EV). (C) Overview of the experimental design for analysis of axonal outgrowth after inhibition of selected axonal miRNAs (D) Representative images of neurons measured after co-transfection with GFP and a specific miR-434-3p inhibitor or non-targeting control (scale bar: 100um). (E) Quantification of axon length in cortical neurons after specific inhibition of miR-434-3p, miR-151-3p and miR-92a showing a decrease in axon length, whereas inhibition of miR-16-5p results in an increase in length of cortical axons. Data presented as % of control expressed in mean±s.e.m, n = 3–6 independent experiments. Student’s t-test, *p-value <0.05.

As a first approach to analyse the miRNA profile in these intra and extracellular compartments we ranked the top 150 miRNAs in each sample by number of reads (Table S3). This analytical approach shows that there is a 73% overlap in the top 100 miRNAs (Fig S3A), a demonstration of the existence of a critical number of miRNAs with high expression levels across all neuronal and EV compartments. In the case of the axon, a closer look at previously published functional data shows that nearly all of the top 20 axonal miRNAs in our list (Table S3 and Fig. 4A) have been reported in profiling studies [52,53,55,56,59,82] and/or analysis of axon development/synaptic function [39,54,83,84]. Moreover, reports of functional relevance extend well-beyond the top 20 miRNAs, for example with miR-16-5p, a miRNA that controls axon outgrowth in superior cervical ganglia axons [57], appearing in position 37 and several known axonal miRNAs also present in the top 100 of our AX list. This initial analysis of miRNA read rankings provides an unbiased approach that strengthens the usefulness and validity of this data set in the prediction of functionally relevant miRNAs.

Beyond the analysis by ‘presence’ within a biological sample, the relative ‘enrichment’ and/or specific localization has been previously used as an analytical step in the identification of relevant miRNAs. This approach requires the comparison of different compartments (i.e. AX vs WC or EV vs WC) and has some important caveats. First, in compartmentalized microfluidic models, the WC compartment contains a proportion of those axons extending from the cell body, and thus a detection of some expression differences between AX and WC might be attenuated or missed. Secondly, important miRNAs with known axonal localization and function might not be shown in ‘enrichment’ datasets due to their associated high expression in somatic compartments, a case observed for two miRNAs previously studied by us, miR-9-5p and miR-26a-5p [24,50], among others. Despite these analytical drawbacks, ‘enrichment’ analysis offers the potential to identify a subset of differentially localized miRNAs with a high probability of functional relevance. As shown in Fig S3A, comparison of the top ranked 100 miRNAs finds that 6–8% of this total are present in only one of the analysed compartments (WC, AX or EVs), and we found several differentially expressed miRNAs in both AX and EVs compared to WC (using stringent cut offs of |Log_2_FC| ≥ 1 and FDR < 0.01) (Fig. 4B and Table S3). These two analytical steps demonstrate that even though most highly expressed miRNAs are present in all relevant compartments, several specific miRNAs accumulate/localize in different subcellular and EV domains, which might determine their properties and contribute to their ability to respond to local cues. As a novel and potentially interesting example, the highly significant enrichment of miR-10a-5p and miR-10b-5p in AX samples compared to WC (Table S3) points towards their potential relevance in axonal function, a fact that has not yet been reported in the literature and probably reflects their relatively low abundance in the overall axonal read rankings.

In the case of EVs, the two-step enrichment analysis shows preferential localization/enrichment of various miRNAs with previously known roles in EVs, mainly of cancer origin, such as miR-126a [85], miR-335-5p [86], miR-421-3p [87], miR-1298 [88], miR-378a-3p and miR-146a-5p [89]. These findings reinforce the strength of our datasets in representing true compartmentalization and supports its use in the identification of not yet recognized miRNAs with important roles in EV-mediated miRNA regulation in neuronal models. Indeed, the majority of the top 50 and 100 most abundant miRNAs in our neuron-derived EVs have been identified in the list of 277 *Mus musculus* EV miRNAs reported in Vesiclepedia (http://microvesicles.org/;) [90], but with the number present in this data resource significantly decreasing in the bottom 50 miRNAs and also in random selections of non-top 100 most abundant miRNAs in our EV samples (Fig S4A). The presence of EV-specific miRNAs in neuronal EVs suggests the existence of a precise sorting mechanism, a process that has received increased attention in cancer models, where key miRNA motifs have been observed to support exosome/EV sorting [91–96]. To explore if these mechanisms operate in our neuronal EVs, we investigated the presence of previously reported miRNA sorting motifs. As shown in Fig S4B-C, the majority (36) of the top 50 most abundant miRNAs in EVs show at least one of the motifs, with complete absence only observed in 14 of those miRNAs. On the other hand, there is a clear decrease in the number of miRNAs with sorting motifs when analysing the least abundant miRNAs in EVs (Fig S4C). These findings suggest how sorting mechanisms reported in cancer cells might be functioning in neuronal models.

### Validation of axonal miRNAs by RT-qPCR and functional axon growth assay

Being the most widely studied sncRNAs, miRNAs allowed us to develop two validation approaches to complement our sequencing datasets, using both quantitative PCR and functional axon growth assays. As shown in Fig S3B, we performed an RT-qPCR miRNA expression array to test the presence in the AX compartment of 21 miRNAs found in the top 100 of sequencing reads from the axon. Overall, miRNAs in the expression array had an average Ct value of 29.9, well below the Ct<35 detection threshold proposed for neuronal miRNAs [55]. Those miRNAs with lower read ranking in the axon sequencing data, could not be detected (Ct>35) in the array, e.g. miR-26b in position 123 of the top 100 miRNAs in the axon samples. In addition to the RT-qPCR arrays, we performed a further validation by RT-qPCR assays of specific miRNAs (Fig S3C-D), including: selected most abundant miRNAs across samples (let-7c-5p, miR-181-5p, miR-26a-5p), the highest miRNA by ranking in the axon (let-7c-5p), and selected most enriched miRNAs in AX vs WC (miR-2137, miR-145a-5p, and miR-10a/10b-5p) and EV vs WC (miR-2137, miR-145a-5p), and which confirmed their relative abundance in RNA-seq data.

To test the capacity of our list of axonal miRNAs to foretell functional relevance in axon development, we carried out a tailored functional screen with selected miRNA inhibitors (Fig. 4C-E). First, we used a specific inhibitor for miR-434-3p, a non-conserved miRNA with high expression in our list of axonal miRNAs but with no functional role yet described in the literature. Inhibition of miR-434-3p showed a significant decrease in axonal length 48 hours after addition compared to non-targeting control (Fig. 4D-E). A similar effect on axon length was observed after inhibition of miR-151-3p, a miRNA that is ranked 28 in our list and which despite its appearance in a previous miRNA screen [56] had no reported axonal function (Fig. 4E). In further confirmatory studies, we showed an increase in axonal length after inhibition of miR-16-5p and a decrease following miR-92a-3p inhibition (Fig. 4E), two miRNAs with long known axonal function [57,97] and ranked within the top 40 in our list. Overall, our axonal growth assay provides robust support for the usefulness of our sequencing data list as a bona-fide predictor of functional role in axons.

### tRNA-derived small RNAs

**Figure 5.**
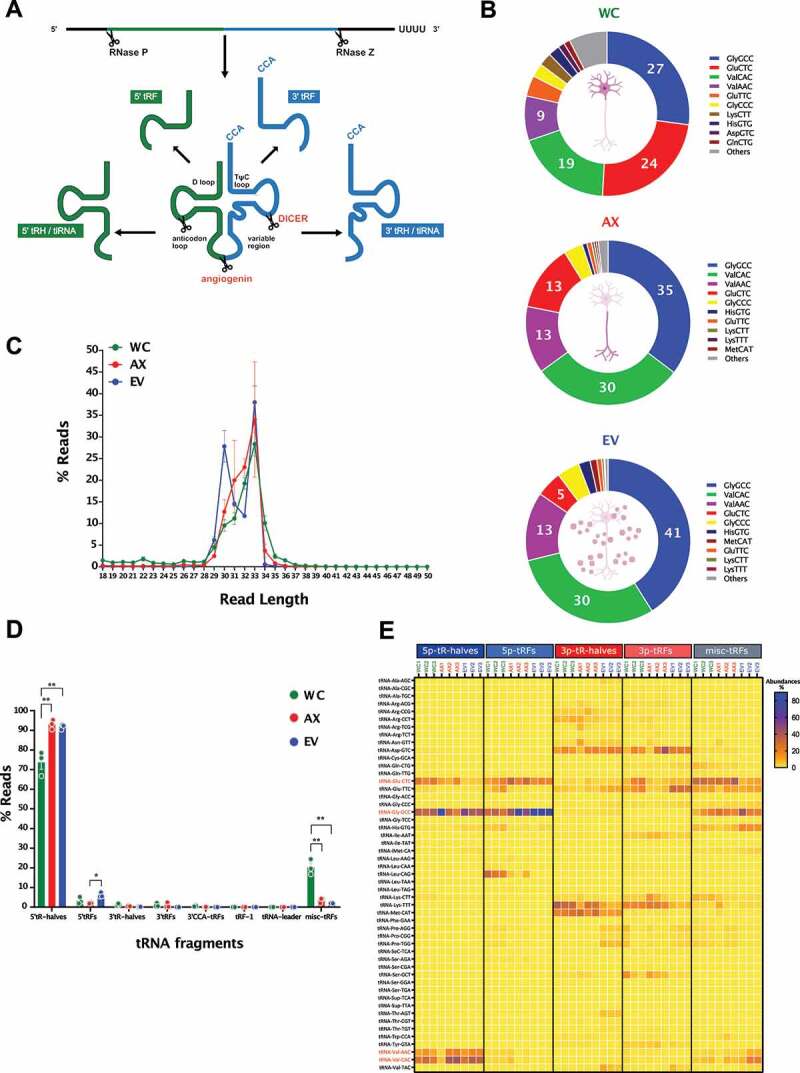
tRNA repertoire in the neuronal subcellular and extracellular compartments. (A) Diagrammatic representation of the biogenesis of tRNA-derived small RNAs (tsRNAs), where mature tRNAs undergo endonuclease cleavage to generate tRNA-derived fragments (3ʹ- and 5ʹ- tRFs) and tRNA halves (3ʹ- and 5ʹ- tRHs). (B) Relative read abundance of parental tRNAs upon tsRNAs mapping in WC, AX and EV shows the most expressed tRNA species in each neuronal compartment (percentage of total tRNA reads). (C) Read length (nt) distribution plot for all tsRNAs (mean±s.e.m.), showing a higher frequency of 33nt long reads in all three of WC, AX and EV samples, but an additional 30nt peak that is predominant in EV samples. (D) Percentage distribution of total reads mapping to each class of tsRNAs in all samples following the unitas annotation workflow (mean ± s.e.m). Comparisons between neuronal compartments demonstrate that 5ʹ-tRHs are the most abundant tsRNAs in all samples and represent a significant higher proportion of AX and EV tsRNAs (~90%) compared to WC (70%). (E) Heatmap of the abundance distribution of tsRNAs present in all WC, AX and EV samples. Data expressed as percentage of total reads per tsRNA class. Highlighted in red are the parental tRNAs generating the most abundant 5ʹ-tRHs: 5ʹ-tRHs-Gly-GCC, 5ʹ-tRHs-Val-AAC, 5ʹ-tRHs-Val-CAC and 5ʹ-tRHs-Glu-CTC. Two-way ANOVA with Tukey’s multiple comparison post-hoc test, * p-value < 0.05; ** p-value < 0.01. Whole Cell (WC), Axon (AX) and Extracellular Vesicles (EV).

Next generation sequencing has accelerated the enumeration and characterization of fragment derived tRNAs (tsRNAs), which far from being random tRNA degradation products, show a biogenesis into 16–35 nucleotide fragments that happens in a highly controlled manner and have been associated with a growing list of biological processes, from cancer to development, [60,61,62]. Although their profiling and functional assessment has gained momentum in a variety of tissues and systems, including neuronal cell lines [98,99], studies in primary neuronal models and/or neuronal tissue have lagged behind, with Jehn et al. [100] only recently showing the high expression of 5ʹ tRNA halves in the primate hippocampus and their potential role in the regulation of gene expression. As shown in Fig. 5A, our findings centred on the 5ʹ and 3ʹ tRNA halves, defined as 5ʹ- or 3ʹ- tRHs but also reported in the literature as 5ʹ- or 3ʹ- tiRNAs, and the smaller 5ʹ and 3ʹ fragments (5ʹ- or 3ʹ- tRFs). In WC samples, approximately 15% of all reads were mapped to tRNAs, a proportion that was dramatically increased to nearly 70% in axons and 90% in EVs (Fig. 2C). Similar to recent findings in mouse embryonic stem cells [101], we found that tsRNA biogenesis appears to be mainly derived from a specific population of full tRNA transcripts, with Gly-GCC, Val-CAC and Val-AAC being the most abundant across all samples, and with cell bodies also having relatively higher levels of Glu-CTC and Glu-TTC (Fig. 5B). As shown in differential expression analysis where tsRNAs are mapped to their parental tRNAs (Fig S5 and Table S4), two clusters of tRNAs appear to concentrate most of the differentially expressed mapped reads, with one group significantly higher in EVs, and another in WC samples. Although AX samples do not show marked clustering of differential expression for tRNAs, they appear more closely associated with those found in WC (Fig S5). This analysis of parental tRNAs provides a first approach in their profiling, but only a more detailed analysis of specific tsRNAs can shed light on their potential relevance and functional significance. Indeed, while all three of WC, AX and EV samples have a marked peak of 33 nt reads for tsRNAs (Fig. 5C), EV samples also show a distinct peak corresponding to 30 nt in length, an indication of differences in tsRNA populations.

Despite advances in sncRNA mapping tools, the specific annotation of tsRNAs can be challenging. As a result, recent work by the Rosenkranz lab [102] developed ‘unitas’, a precise and sensitive universal tool for annotation that can be used for tsRNAs. Using this annotation protocol, we demonstrate that 5ʹ-tRHs are by far the most abundant tsRNAs in all samples analysed (Fig. 5D and Table S5), but with significantly higher levels observed in AX and EVs (~ 90%) compared to WC (~ 70%). The smaller 5ʹ-tRFs are present at relatively low levels in all samples, but with EVs still showing a slightly higher percentage of reads (Fig. 5D). Among the highly prevalent 5ʹ-tRHs, reads are dominated by 5ʹ-tRHs-Gly-GCC, 5ʹ-tRHs-Val-AAC, 5ʹ-tRHs-Val-CAC and 5ʹ-tRHs-Glu-CTC, which show largely similar levels in all samples, and with only the latter having relatively low expression in EVs (Fig. 5E). As further validation, we performed an RT-qPCR for 5ʹ-tRHs-Gly-GCC, 5ʹ-tRHs-Val-AAC, 5ʹ-tRHs-Val-CAC, as shown previously for miRNAs, confirming their relative compartment abundance as determined by RNA-seq (Fig S3E). Interestingly, both 5ʹ-tRHs-Glu-CTC and 5ʹ-tRHs-Gly-GCC have been previously shown to play roles in various cellular functions and are highly expressed in the primate hippocampus [100], where they were proposed to fine-tune hippocampal gene expression. Notwithstanding the high expression of these 5ʹ-tRHs among mapped tsRNAs, the much smaller percentage of 3ʹ-tRHs is centred around 3ʹ-tRHs-Asp-GTC, 3ʹ-tRHs-Lys-TTT and 3ʹ-tRHs-Met-CAT (Fig. 5E). Interestingly, the miscellaneous category of tRFs is significantly increased in WC compared to AX and EVs, despite it being dominated by 3ʹ-tRFs for Glu-CTC and Glu-GCC, which are increased for their 5ʹ arms in the AX and EV samples. The latter finding might provide an indication not just of the specific localization of tsRNAs, but also of processing steps occurring in the WC before specific axonal transport and/or EV sorting.

### snoRNAs, snRNAs and other sncRNAs

**Figure 6.**
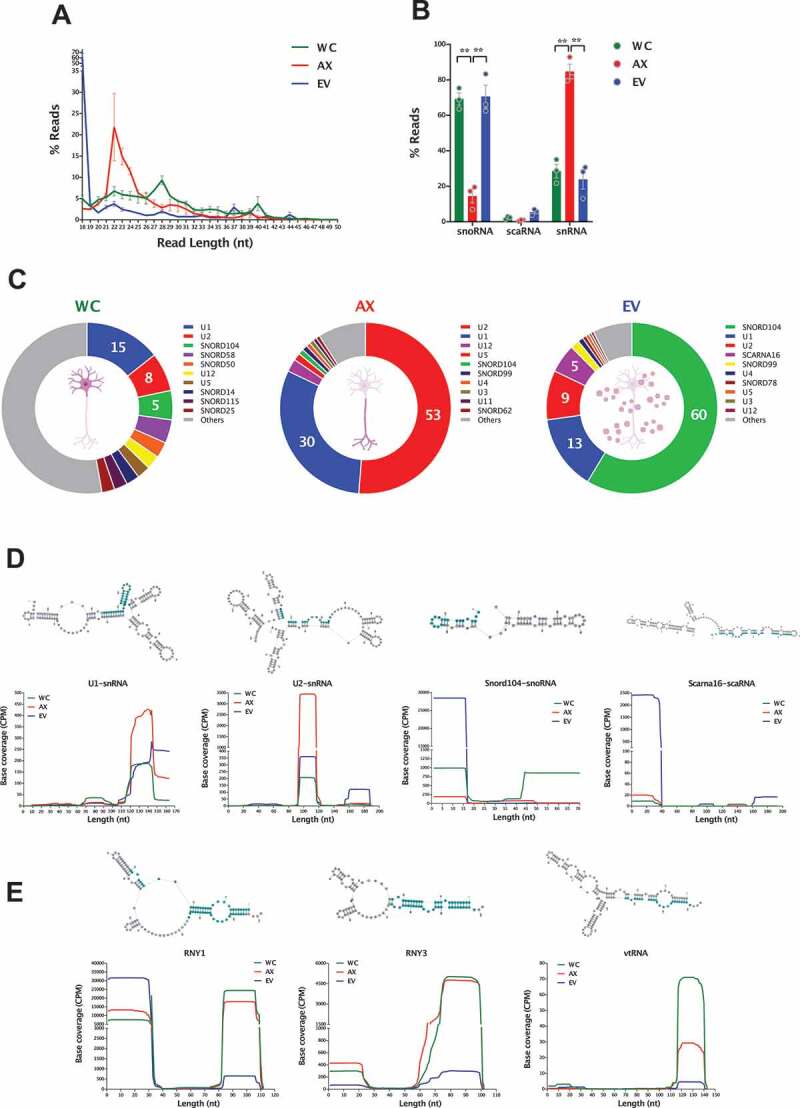
sRNAs (snoRNAs, scaRNAs and snRNAs) in the neuronal subcellular and extracellular compartments. (A) Read length (nt) distribution plot of reads mapping to parental sno/scaRNAs and snRNAs shows the distinct profile between neuronal compartments (mean ± s.e.m.), with a marked 22nt peak in AX samples, which is largely absent in WC and EVs. (B) Mean percentage distribution of total reads mapping to each class of sRNAs investigated in all WC, AX and EV samples (mean ± s.e.m.). Comparisons between neuronal compartments show that snoRNA-derived small RNAs compose the majority of sRNA reads in WC and EV samples (~70%), whereas snRNA fragments are the most abundant class in the AX. (C) Relative abundances of those RNA fragments mapping to parental sno/scaRNAs and snRNAs in WC, AX and EV showing the most expressed in each neuronal compartment. Data expressed as percentage of total sno/scaRNAs and snRNA reads. (D) Schematic secondary structure of consensus sequence (top) and base coverage plots of the most expressed parental sno/scaRNAs and snRNAs (bottom) reveal specific processing into shorter well-defined RNA fragments, whose expression pattern differs in the subcellular and EV compartments. (E) Schematic secondary structure of consensus sequence (top) and base coverage plots of the most expressed parental YRNAs and vtRNAs (bottom) also reveal processing into shorter well-defined RNA fragments. Note how the distinct processing patterns are dependent on subcellular and extracellular localization. Most abundant processed fragments are highlighted in blue in the secondary structures. Two-way ANOVA with Tukey’s multiple comparison post-hoc test, * p-value < 0.05; ** p-value < 0.01. Whole Cell (WC), Axon (AX) and Extracellular Vesicles (EV).

Known for their role in the modifications of ribosomal and spliceosomal RNAs, snoRNAs are also being associated with additional non-canonical functions in the cytoplasm, which are mainly linked to stable shorter fragments defined as sdRNAs (snoRNA-derived RNAs) [103]. In similar fashion, the mapping of sequence reads to snRNAs, which are traditionally known to be involved in splicing and the spliceosome complex, have shown specific peaks in base coverage plots that are indicative of selective cleavage [104]. In our neuronal samples, we found that reads mapping to snoRNAs or snRNAs have clear differences in length distribution when comparing the different subcellular and EV compartments (Fig. 6A). Indeed, the percentage of reads that correspond to sdRNAs are a major component of both WC and EV samples, but this is reversed in the axon, where instead we observe a significant increase in the localization of fragments derived from snRNAs (Fig. 6B and Table S6). In order to address the origin of these small RNAs we first grouped them to their parental sno/scaRNA or snRNA defined by RFAM, and we detect clear differences in the sub-cellular and EV relative abundance, with fragments corresponding to U1 and U2 snRNAs dominating WC and AX samples, but with AX showing a dramatic increase in their relative abundance (Fig. 6C and Table S6). Crucially, of those fragments that have most reads, the base coverage patterns show clear processing into short and defined small RNAs (Fig. 6D). In the case of snRNAs in the axon, there is a clear accumulation of fragments derived from U1 and U2 that correspond to precisely processed ~20-25 nt small RNAs (Fig. 6D), and which are significantly increased when compared to both WC and EV samples. As shown in the plots displaying the read base coverage along consensus sequence and schematic secondary structures, U1 and particularly U2 derived fragments are concentrated along precisely processed regions that include Sm sequences [104]. Among the reads that map to snoRNAs, the 5ʹ end of snord104 is the most abundant sdRNA in both the WC and EV samples, accumulating over half (60%) of the sno/snRNA derived fragments in the latter. Finally, EV samples also show a relatively high abundance of the scaRNA16 fragment (Fig. 6D).

For Y RNAs, we also observe specific processing patterns that are selectively allocated to the different subcellular and EV compartments. As seen in the base coverage plots in Fig. 6E, we detect two main fragments of ~30 nt derived from the 5ʹ and 3ʹ end of RNY1, with EVs having a significant preference for the 5ʹ arm rather than the 3ʹ. In the case of RNY 3, the 3ʹ fragments are mainly localized to AX and WC, with low levels of expression in EVs. For vtRNAs, we also find a similar arm preference, with the main fragment detected corresponding to a unique ~30 nt small RNA that is largely absent from EV samples. Overall, the Y RNAs provide an interesting glimpse of the specific processing, arm selection and preferential sorting of small RNAs derived from full-length sequences.

The piRNAs are among the other sncRNAs that have been lately reported to be expressed in neurons and brain tissues [105,106], and our initial quantification using piRNAs genomes coordinates from piRNABank database [107] also found a significant number of possible piRNA identities. However, recent studies have clearly established that a considerable number of sequences in piRNA databases have significant overlapping identity to other ncRNAs [108,109]. Crucially, these sequences appear to account for the vast majority of piRNAs described in the mouse brain [106,109]. For this reason, we followed a filtering procedure described by Godoy et al., 2018, which involved the exclusion of ambiguously mapped piRNA reads from our final list (~97% of reads initially mapped to piRNAs were thus excluded). From this strict analytical workflow, only 5 piRNAs showed a significant number of reads in our samples, including DQ540862, DQ541470, DQ696831 and DQ540412 (Table S1). Of these, the first three show relatively low levels in EV samples compared to WC, which might suggest an active mechanism of selective sorting, as shown for other sncRNAs in our study. On the other hand, DQ540412 shows comparable, if not higher levels of expression in EVs.

### sncRNA profiling from the axoplasm of adult axons in vivo

The relevance of sncRNA localization to neuronal compartments is not limited to developmental stages. Indeed, several mRNA transcripts and their functional roles have been identified and investigated in mature axons [8,17,21,110], supporting the idea that RNA localization to the axon is necessary for axon maintenance and normal neuron physiology throughout life.

In a recent study, the Sotelo-Silveira lab used a modified axon micro-dissection method to profile the mRNAs populating the pure cytosolic fraction (axoplasm) of mature motor neurons *in* *vivo* [11]. Despite the technical challenges, in particular the difficulty in obtaining pure axonal cytoplasm from *in* *vivo* nerves and the very low RNA yield, this method opens the window to the highly diverse RNA population of mature myelinated axons in their normal microenvironment, without the confounding RNA originating from glial cells [11]. Using the same experimental approach, we profiled the sncRNAome of axoplasm preparations from both rat myelinated motor and sensory neurons, with the aim to determine the sncRNAs biotypes and specific sncRNA species present in the mature axon and compare to those found in developing axons *in vitro*.

**Figure 7.**
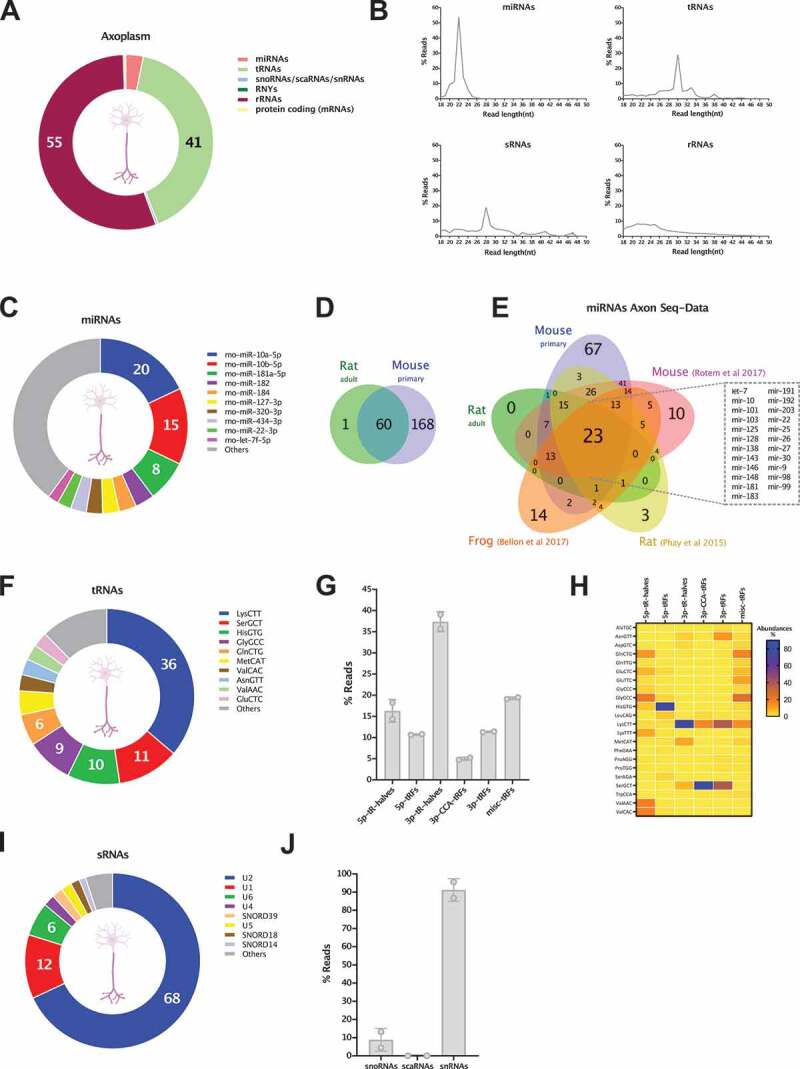
sncRNA profiling of the axoplasm from adult axons. (A) Relative read abundance of each sncRNA biotype in the peripheral nerve’s axoplasm (percentage of total sncRNA reads). (B) Read length (nt) distribution plot of reads mapping to miRNAs, tRNAs, sno/snRNAs (sRNAs) and rRNAs. (C) Relative read abundance of specific miRNAs showing the most expressed in axoplasm samples. Data expressed as percentage of total miRNA reads. (D) Venn diagram illustrates the very high overlap of individual miRNAs detected in the *in* *vivo* rat axoplasm (miRNAs ≥ 5 reads) and the *in* *vitro* mouse axons (miRNAs ≥ 50 CPM, which is equivalent to ≥ 10 reads). To compare miRNAs of different species only the precursor’s base identification was considered in each case. (E) Venn diagram displaying the overlap of miRNAs detected in our two axon sncRNA-seq datasets (*in* *vivo* rat axoplasm and mouse *in* *vitro* axons) and the three currently available RNA-seq axon datasets, *in* *vitro* rat motor axon, frog retinal ganglion cells axons and rat sciatic nerves. As above, only the precursor’s base identification was considered in each case. This analysis reveals a core of 23 axonal miRNAs present across all neuronal types, both in *in* *vivo* and *in* *vitro* axons. (F) Relative read abundance of parental tRNAs shows the most expressed parental tRNA species in axoplasm. Data expressed as percentage of total tRNA reads. (G) Percentage distribution of total reads mapping to each class of tRNA-derived small RNA (tsRNAs), following the unitas annotation workflow (mean ± s.e.m). (H) Heatmap of the distribution of each tsRNA class present in the axoplasm with at least 1% of relative abundance. Data expressed as percentage of total reads from tsRNA class. (I) Relative read abundance of parental sno/scaRNAs and snRNAs shows the most expressed in axoplasm samples. Data expressed as percentage of total sno/scaRNAs and snRNAs reads. (J) Percentage distribution of total reads mapping to each class of sRNAs (snoRNAs/scaRNAs/snRNAs) investigated in axoplasm samples (mean ± s.e.m.).

We obtained axoplasm preparations from both rat ventral root and dorsal root nerves, and separate sequencing datasets for the individual samples can be found in Table S7. In subsequent analytical steps we decided to combine the sncRNAseq datasets from both axoplasm samples to achieve a more representative picture of the sncRNAs present in mature axons. Sequencing reads from the axoplasm samples were mapped to the rat genome to reveal a diverse sncRNA population corresponding to specific biotypes (Fig. 7A and Table S7). Although no reads were mapped to piRNAs, we found that miRNAs, rRNA and tRNA fragments compose the vast majority of detected RNA biotypes. Interestingly, the percentage of reads mapping to miRNAs (4.2%) and tsRNAs (41%) in mature axoplasm samples resembles the ratio observed in developing axons (AX), with the former making only a small percentage of total. Unlike *in vitro* axons, the most abundant biotype in mature axon samples is that of rRNA fragments, which make 55% of total mapped reads. Although differences in sample preparation and sequencing coverage from ultra-low input samples might be expected, the overall distribution of RNA biotypes appears to follow similar patterns in both developmental and mature conditions, supporting the view that sncRNAs are relevant regulatory players in both developing and mature axons. A closer look at the size length distribution of the mapped features reveals discrete read abundance peaks for the main sncRNA biotypes detected. This is particularly prominent for miRNAs with a 22nt peak corresponding to mature miRNA sequences and for tsRNAs which exhibit a 30nt peak consistent with tRNA halves (Fig. 7B).

Further analysis of specific sncRNA biotypes identified and ranked the miRNAs detected in the axoplasm by read abundance (Fig. 7C). Interestingly, miR-10a/b-5p, the top miRNAs in the *in* *vitro* AX vs WC enrichment analysis (see above), correspond to 35% of all miRNA reads in the nerve axoplasm. Upon overlapping the miRNA species detected in the rat axoplasm and mouse axons, all but one of the miRNAs detected in the axoplasm are also present in mouse axons *in vitro* (minimum average of 5 and 10 reads for axoplasm and cortical samples respectively, see methods and Fig. 7D). Moreover, 80% of the 60 miRNAs present in both samples of axoplasm are ranked in the top 100 mouse axon (AX) miRNAs, while Pearson correlation analysis of miRNA expression levels shows the strongest correlation between axoplasm and AX samples (r = 0.7, p value 2.20 E-16), compared to axoplasm and WC (r = 0.47, p value 1.00 E-08). This marked similarity between axonal miRNA datasets originating from developing and mature axons from distinct neuronal types (cortical primary neurons, motor and sensory neurons) further supports the capacity of this dataset to predict those miRNAs crucial to axon physiology. With this aim, we compiled the miRNA profiles from the current five sncRNA sequencing datasets available for axons, these being our own rat axoplasm and mouse primary neurons, plus the *Xenopus laevis in* *vivo* retinal gangion cells [59], rat sciatic nerves [111] and cultured spinal cord neurons [82]. For this analysis, and in order to compare miRNAs of different species, only the precursor’s base identification was considered, and a minimum of 5 reads average was used as inclusion criteria for the previously published studies. As illustrated by the Venn diagram in Fig. 7E, this analysis allowed us to define a subset of 23 conserved miRNAs detected across the axonal samples, including different neuronal subtypes, species and both in *in vivo* and *in vitro* models. Interestingly, this core group of miRNAs includes well known axonal miRNAs, such as miR-9, miR-128, miR-181 and miR-26 [24,50,84,112], and miR-10, the most abundant in our axoplasm samples and most enriched in cortical axons. Overall, this constitutes a significant step forward towards determining an axonal miRNA signature.

For the tRNA analysis, we used the workflow previously devised for AX samples. After mapping the sequencing reads to parental tRNAs, the read distribution analysis revealed that reads corresponding to Lys-CTT make 36% of the axoplasm’s tRNA reads, followed by Ser-GCT (11%) and His-GTG (10%). The most abundant tRNA reads in the *in* *vitro* AX samples, reads corresponding to Gly-GCC, Val-CAC and Val-AAC, were far less abundant in mature axoplasm samples (Fig. 7F), potentially reflecting differences between the neuronal types and/or a functional shift from developing to mature axons. When moving to the specific analysis of tsRNAs, 3ʹ-tRHs, nearly absent in developing cortical axons (Fig. 5D), represent ~40% of all tsRNA reads in the nerve (Fig. 7G), including the highly abundant 3ʹ-tRHs of Lys-CTT (Fig. 7H). Conversely, 5ʹ-tRHs, dominant in developing axons (~90% of tsRNA reads), were far less abundant in the axoplasm, at approximately 15% of total (Fig. 7G). This redistribution of tsRNAs abundance in axoplasm includes the increase in the proportion of other 3ʹ tRNA-derived fragments, 3ʹ-tRFs and, to a less extent, 3ʹ-CCA-tRFs, unveiling a 3ʹ tRNA preference in these samples (Fig. 7F-H).

Finally, we analysed the distribution of reads mapping to sRNA features: snoRNAs, scaRNA and snRNAs. Similar to *in* *vitro* axons, reads corresponding to fragments of U2 compose the majority of total sRNA reads detected in the axoplasm (68%), followed by U1 (12% of sRNA reads) and U6 (6% of sRNA reads) (Fig. 7I). The most abundant snoRNA, SNORD39 represents 0.05% of sRNA reads and, in agreement with the low levels found in *in* *vitro* axons, scaRNAs were not detected in our axoplasm samples. Interestingly, the proportion of the analysed sRNA features closely resembles the one observed for *in* *vitro* AX samples, with a striking predominance of reads corresponding to snRNAs (95.5%).

### Functional prediction analysis for most abundant miRNAs and tsRNAs in axons and EVs

To investigate the functional implications of the compartmentalized distribution of sncRNAs, we performed a pathway analysis of the most abundant miRNAs and tsRNAs in axons and EVs. TargetScan gene predictions for the top 10 most expressed miRNAs were obtained, and mirPath v1.3 [113] analysis was used to retrieve those KEGG pathways significantly overrepresented in AX miRNA target gene lists. The resulting list includes neuronal and growth-related processes, from cancer signalling to axon guidance, a prediction pattern that is also observed when investigating the signature of 23 axonal miRNAs which are common to several axon small RNA independent studies (Fig S6A).

Extending this approach to the 10 most abundant miRNAs in EVs, we found that growth and cell mechanisms relevant to developing neurons are also present, including axon guidance, ECM-receptor interaction, proteoglycans and endocytosis (Fig S6B). Indeed, the majority (40) of KEGG pathways predicted for the most abundant miRNAs in AX and EVs are common to both (Fig S6C), suggesting a developmental stage where axonal and EV pathways associated with miRNA regulation are geared towards active axonal growth and the establishment of neuronal connectivity.

To date, the functional prediction of tsRNAs is not as well-defined as that of miRNAs. However, given how gene silencing has been proposed as one of their key roles, recent studies applied miRNA target identification tools to the analysis of tsRNA function [114–116]. Although partial in the consideration of functional mechanisms, we took this silencing mechanism approach to investigate the predicted pathways of the most expressed tsRNA sequences in cortical axons and EVs, 5ʹ-tRH-Gly-GCC and 5ʹ-tRH-Val-AAC/CAC (Fig S6D), and the two most expressed in axoplasm samples, 3ʹ-tRH-Lys-CTT and 3ʹ-tRH-Ser-GCT (Fig S6E). While these are only predictive approximations to the cellular mechanisms at play, they provide a general picture of the functional implications of specific sncRNA localization, one that offers local molecular flexibility to neuronal network development and function.

## Discussion

The discovery of regulatory sncRNAs has led to major advances in the investigation of gene expression, while simultaneously providing novel mechanistic insight in the understanding of neuron connectivity and brain function. Unlike long non-coding RNAs, which act as scaffolds or decoys for other molecular interaction partners, the function of sncRNAs has been normally associated with the provision of target guidance and/or processing properties to multi protein effector complexes. However, beyond these broad similarities in mechanism, and the differences in biogenesis and function that exist, what sncRNAs ultimately offer is the increased capacity for much-needed spatiotemporal control of gene expression in the nervous system, either by their specific subcellular localization and/or via trans-cellular communication [28,44].

The precise identification of sncRNA types in recent years has been coupled to the overall increase in high throughput sequencing technologies and bioinformatics tools that allowed a detailed exploration of their repertoire [104,117]. Although early reports tended to focus on major sncRNA classes, such as miRNAs, in subsequent approaches unclassified reads have emerged from being interpreted as random degradation products to be defined as novel sncRNA families derived from previously well characterized structural sncRNAs, such as tRNAs, snoRNAs and snRNAs [65,104]. Our data provides support to the evolving concept that precisely processed sncRNAs can be biologically relevant RNA fragments rather than the product of non-specific degradation. In effect, we show that across most sncRNAs, and in particular tRNAs and sn/snoRNAs, reads are accurately mapped to specific regions of primary and/or secondary structure with special motifs. Moreover, detected reads for these small RNAs are relatively high, while specific fragments from the same primary transcript are differentially localized and regulated in sub-cellular and extracellular compartments. Crucially, we found key differences in the preference for specific fragments between compartments but also between developing and mature axons, for example, with 5ʹtRHs predominantly expressed in the former and a shift in preponderance of 3ʹtRHs in adult axons. The question of why sncRNAs can manifest this variety of terminal specific processing might be linked to the association to RNAs involved in fundamental and ancient core processes, such as translation, but the list of proposed functional roles has continuously expanded in recent years [65,118]. Importantly, even if the mechanistic role for these RNA fragments does not involve translation regulation by direct mRNA targeting (as with miRNAs), they might act as specific molecular switches by preventing or promoting RNA interactions in ribonucleoprotein complexes. Overall, our study in primary and mature neurons contributes towards an emerging picture where known non-coding RNAs not only function as single transcript units but can act as precursors for other small RNAs with specific localization, sorting and functional mechanisms, providing a first profile of the highly complex landscape of sncRNAs in different compartments of rodent CNS neurons, their released EVs and peripheral mature axons.

### sncRNA localization and selective incorporation

As membrane enclosed nanoparticles, EVs have emerged as a novel information delivery system in most cell communication processes. In work that has so far been focused on plasma, cancer and other non-neuronal cells such as astrocytes, the RNA cargo of EVs has started to be unravelled, showing a complex landscape of ‘traditional’ sncRNAs and derived fragments. Despite these advances, the cellular mechanisms involved in trafficking and sorting remain less well-understood [119], while its role in neuron-to-neuron communication has remained largely unexplored until recently [120,121]. The random versus selective incorporation of RNAs into EVs has also been a topic of intense debate in recent years [122,123] and although the RNAs present in EVs should reflect the type and physiological state of the source cells, they can also differ significantly from their cellular RNA composition [27]. Here, we performed a comprehensive evaluation of all RNA classes/biotypes to provide strong evidence of the selective distribution of sncRNAs along subcellular neuronal domains (WC vs AX) and into EVs. These results support a cellular process in which specifically processed sncRNAs undergo both selective transport into the axon and/or incorporation into EVs, but the precise mechanism and distribution appears to be highly dependent on the particular sncRNA biotype and the presence of specific motifs.

### miRNA profiling in axons and EVs

To date, identification of sncRNAs in neuronal subcellular compartments has been largely focused on peripheral/sensory neurons and miRNAs, with most studies using microarray analysis and RT-PCR screens to show a large and heterogenous population of axonal miRNAs [52,55,56,82]. In cortical neurons, profiling of axonal miRNAs was previously reported using a culture method termed ‘neuron ball’ [53] to isolate distal axon vs somatic RNA, and a qRT-PCR assay system to identify a set of axon-enriched miRNAs. The profiling studies were also accompanied by several papers that addressed the functional role of specific axonal miRNAs in multiple experimental systems, providing strong support for the link between sub-cellular localization and functional properties [39,44]. As addressed in the recent review by Corradi and Baudet [44], initial attempts at identifying a unique axonal miRNA signature have been hampered by the relatively low number of sequencing studies available. Moreover, differences in species, methodological approaches and enrichment vs abundance analyses means that sometimes comparisons between datasets are difficult to establish. Results from our study, however, confirm the existence of core miRNAs with well-established axonal function/localization among our top abundant miRNAs. This is particularly notable in the fact that nearly all the miRNAs detected in the mature axons, are also in the top 100 ranks for miRNAs in developing axons from cortical primary neurons. By analysing our two axonal datasets (*in vitro* cortical and adult axons) together with currently published sequencing data from axonal samples (including mouse and *Xenopus laevis*), we identify a core miRNA signature of 23 miRNAs, a list that includes several well-established axonal miRNAs and also those identified as part of the synaptic sncRNAome [54]. Particularly important in this comparison is the fact that mature axons keep a core selection of those miRNAs found in early neurons, further supporting the view that precise regulation of local axonal translation is not an exclusively developmental phenomenon.

Unlike studies where axonal samples cannot be matched to a corresponding whole-cell source, the use of microfluidic chambers allowed us to perform enrichment analysis comparing axonal vs whole-cell distributions. This meant we could identify some miRNAs with relatively low expression levels but with highly specific axonal localization. The main example of this selective approach is miR-10a-5p, which is not only the most differentially localized miRNA in the axons of primary cortical neurons, but also the most abundant miRNA in the axoplasm of mature axons. The identification of miR-10 as a potentially relevant miRNA, but one for which no previous association with axonal function has been reported, highlights the importance of comparing multiple analytical workflows in the analysis of RNA-seq data in order to unravel the true meaning of sncRNA profiling.

### tRNA-derived small RNAs

It is now well accepted that tsRNAs can be produced constitutively and can mediate gene regulation [100,124,125]. Initially characterized as functional sncRNAs mainly in the context of cell cycle propagation and proliferation in cancer cells [126,127], subsequent studies have linked tsRNAs, and in particular angiogenin-processed ones, to important roles in neuron survival [99,127] and neuro-developmental disorders [128]. Beyond this still incomplete account of their cellular function, one of the intriguing observations of tsRNAs is their association with EVs [26,129], and the recently described biogenesis in the extracellular space [130]. Our data supports the observation that tsRNAs are fundamental components of both the axonal and EV RNAome, providing additional new evidence for their specific localization in neuronal sub-cellular domains, both in developing and adult axons.

Previous studies on tsRNA expression in mammals found a clear difference between primates, which have relatively high expression, and pigs and rodents, with 10% and 3% of mapped reads corresponding to tsRNAs [100]. Our findings in WC neuronal samples support this relatively low level of mapped reads corresponding to tsRNAs. However, we show how when analysing specific sub-cellular and extracellular compartments, neurons present much higher levels of tsRNAs. Indeed, the striking increase in specific tsRNAs in the EVs and axons, both at developmental and mature stages, suggests a specific role in the regulation of neuronal connectivity. In effect, beyond efforts to elucidate their cellular and sub-cellular profiling, studies exploring the mechanistic nature of tsRNA biology have pointed towards two main processes: sequence-specific post-transcriptional silencing and/or global translational repression. For the latter, Ivanov et al. [99] showed how angiogenin generated 5ʹ-tRHs (tiRNAs) cooperate with translational repressor YB-1 to displace eIF4F from capped mRNA and inhibit translation initiation, while also able to assemble unique G-quadruplex (G4) structures required for translation inhibition. In addition to this disruption of translation machinery, tsRNA mediated target silencing via AGO2 has also been proposed in eukaryotic cells [127]. It is thus becoming clear how small RNAs that are processed from larger non-coding RNAs, but distinct from miRNAs, such as tRNAs and snoRNAs, can be associated with argonaute proteins and play a role in RISC mediated posttranscriptional gene regulation [131,132], although a role for some of them in AGO1-dependent chromatin remodelling has also been proposed [131]. Whether the observed population shift of tsRNAs, from 5ʹ-tsRNAs in primary cortical neurons to 3ʹ-tsRNAs in peripheral axons is linked to the maturation of the axon in the adult animal, remains to be investigated.

### snoRNAs, snRNAs and other sncRNAs

Similar to the developments observed in tRNA biology in the last decade, RNA fragments derived from other sncRNAs have also gained increased attention, with our findings confirming the existence of differential distribution among the neuronal sub-cellular and EV domains. Unlike the axon, which has a preponderance of snRNA derived fragments, both WC and EVs show a higher prevalence of sdRNAs, with the ~15 nt 5ʹ end of SNORD104 making almost 60% of the EVs sRNAs. Interestingly, and despite the fact that snoRNAs are largely more common in cells than in EVs, SNORD104 and SNORD99, which are both detected in our neuron derived EVs, have been previously found to be more abundant in exosomes isolated form endothelial cells than in their cells of origin [133]. The regulatory potential of these sdRNA fragments has only started to be unravelled, showing differential expression upon stress conditions in yeast [103] and in tumour development, where SNORD78 and its derived sdRNA was shown to be significantly increased in patients that developed metastatic disease [134]. The association of these sdRNAs with cell processes linked to active cell growth in metastatic cells might also explain their presence in EVs of developing neurons, suggesting a common signalling approach to two different cellular phenomena. In addition to sdRNAs, we also observed that axonal samples in developing cortical neurons presented a significantly higher preponderance of snRNA derived fragments, and in particular those processed from U2, which comprises 51% of all sRNA reads. Interestingly, precisely the same fragment of U2 is also present among the snRNAs in mature axons.

Among the other sncRNAs present in our samples, we focused our attention on small non-coding Y RNAs, which are well-conserved in all vertebrates and have two copies present in the mouse genome (RNY1 and 3). As single-unit transcripts of ~ 100 nt, the 5ʹ and 3ʹ ends hybridize to form predominantly double stranded upper and lower stem domains with an internal, more variable, loop [135]. This modular structure is essential to the understanding of their binding properties and functions, which have been linked to RNA processing, stability and DNA replication [135]. More recently, 5ʹ Y RNA derived fragments (s-RNYs) were shown to regulate cell death and inflammation in human monocytes/macrophages via recognition by toll-like receptor 7, upregulation of cleaved caspase 3 and downregulation of IkBa [136]. The presence of s-RNYs has also been detected in proliferating cells, both cancerous and non-cancerous, and in the brain [135]. Although neuron-specific mechanisms for s-RNYs have not been fully explored, we found that different 3ʹ and 5ʹ fragments of RNY1 and RNY3 are expressed in neuronal compartments, which suggests the intriguing possibility that rather than degradation products of abundant Y RNAS, s-RNYs are part of local regulatory processes controlling neuron function.

Unlike Y RNAs and vtRNAs, which derive from only few numbers of gene copies, PIWI coding regions are poorly conserved and complex in origin and organization. Primarily identified in germ cells and associated with the defence of germline genome against transposon mobilization [137], their presence and capacity for regulation has been also reported in other tissues, including roles as repressors of axon regeneration in C. elegans [138]. Despite these advances, the understanding of their non-transposon function remains technically and intellectually challenging, with recent reports also highlighting the problems associated with piRNA databases [108,109], which might have confounded recent profiling efforts. In effect, a substantial number of piRNA-mapped reads also mapped to other RNA biotypes, supporting the view that mapping to a given piRNA database should not be considered as sufficient proof for their presence [109]. When the analysis was confined to those piRNA sequences that did not map to other biotypes we found only 5 piRNAs with significant number of reads. Despite the reduction in those piRNAs mapped, their expression still suggests a functional role in neuronal function.

From the early stages of molecular biology research it was known that eukaryotic gene sequences can superimpose multiple layers of information. Although this observation was initially derived from studies of transcription initiation, termination and splicing, more recent advances in modern sequencing technologies have made apparent that a single transcript of non-coding RNA can use shared sequences in multiple ways that fulfil a wide spectrum of fundamentally different cellular functions [117]. This provides cells, and neurons in particular, with a complex and dynamic set of molecular tools that can be used towards the precise spatiotemporal control of neuronal function and communication. Our data throws light into the complex landscape of sncRNAs in neuronal models, both at sub-cellular and extracellular level, prompting the need for further studies into their localization and functional mechanisms.

## Methods

### Animals

Mice (C57BL/6) were housed, bred and sacrificed (Schedule 1) in compliance with the ethics and animal welfare in accordance with the Animal (Scientific Procedures) Act 1986, in place in the University of Nottingham, UK. Rats (Sprague–Dawley) were housed, bred and sacrificed in strict accordance with the Comité de Ética en el Uso de Animales of Instituto de Investigaciones Biológicas Clemente Estable (CEUA-IIBCE) under law 18.611 of the República Oriental del Uruguay. The specific protocol was approved by the CEUA-IIBCE (experimental protocol N°005/05/2012).

### Primary cortical neuron cultures

Embryonic day 16.5 (E16.5) embryos from timed-pregnant C57BL/6 mice were used for the isolation of primary cortical neurons, which were obtained as described previously [24]. Briefly, after dissection of the cortical region and meninges separation, the tissue was incubated in Hanks Balanced Salt Solution (HBSS, Ca^2+^ and Mg^2+^-free; Gibco) with 1 mg/ml trypsin and 5 mg/ml DNAse I (Sigma-Aldrich) at 37°C for 30ʹ followed by the addition of 0.05% (v/v) soybean trypsin inhibitor (Sigma-Aldrich). Upon mechanical dissociation of the tissue, dissociated cells were resuspended in Neurobasal media (Invitrogen) supplemented with 1% GlutaMax and 2% B-27 (Gibco), and seeded onto poly-L-ornithine (PLO) coated 6-well plates (0.05 mg/mL overnight; Sigma).

### Compartmentalized neuronal culture in microfluidic chambers

Two-channel microfluidic devices with a 150 μm microgroove barrier across channels (SND150, Xona microfluidics) were used to separate axons of cortical neurons from their somato-dendritic compartment. The devices were prepared as previously described [24]. Briefly, the devices were sterilized in 70% (v/v) ethanol, incubated in HBSS media overnight for removal of potentially toxic manufacturing by-products, washed in sterile water, and mounted onto well-dried PLO-coated (0.05% (w/v), overnight) 35 mm dishes (Nunc, ThermoFisher). Dissociated cortical neurons were plated into the designated somatodendritic compartment at a seeding density of 4.0 × 10^6^ cells/ml and incubated for 30ʹ (37°C, 5% CO_2_) to allow for cell attachment. The devices’ reservoirs were then topped up with supplemented Neurobasal media and incubated at 37°C, 5% CO_2_. Axons were allowed to extend and cross the microgrooves to the axonal channel. In this model, as the neuronal culture develops, both dendrites and axons grow in the seeding channel (whole cell side), whereas only axons cross the microgrooves to the second channel, designated as axon side (Fig. 1A-C). Culture media was replenished 24 h after plating and every 3 days thereafter.

### Extracellular vesicle isolation

The conditioned media of primary cortical neurons cultured 9 days *in* *vitro* in four 6-well plates (seeding density 1.75 × 10^5^ cells/cm^2^) was collected per individual preparation. Pooled culture media (~48 mL) underwent filtration using a 10 K MWCO centrifugal concentrator (Pierce) for 30 min/4000 g to a final volume of 500uL. Extracellular vesicle (EV) fractions were further isolated by size-exclusion chromatography using commercially available sepharose columns (pore 70 nm, qEV IZON), in which the EV fraction in the media is separated by gravity flow (Fig. 1B), using Ca^2+^/Mg^2+^-free PBS as elution buffer, in accordance to manufacturer’s procedure to isolate small EVs <200 nm and within the range considered to represent exosomes [67]. This method attempts minimal disturbance of vesicle size and content by avoiding ultracentifugation forces, thus preserving EV integrity and, importantly, their biological activity [72].

### Extracellular vesicle characterization

Following EV isolation, the EV fraction was measured by nanoparticle tracking analysis (NTA) to quantify EV size distribution and concentration. NTA was performed using the PMX 110 ZetaView (Particle Metrix, Meerbusch, Germany). Laser light scattering was used to visualize the Brownian motion of each traced EV and tracked over time to calculate particle size using the Stokes-Einstein equation to determine the translational diffusion constant. The parameters for all NTA measurements were as follows: sensitivity 85, shutter value 70 (corresponding to an exposure time of 15 ms) with a frame rate of 30 frames per second. For all samples 3 technical assessments were carried out. For EV immunoblot analysis, PBS-washed cortical primary neurons and isolated EVs were lysed directly in gel loading buffer (0.15 M Tris, 8 M Urea, 2.5% SDS, 20% glycerol, 10% 2-mercaptoethanol, 3% DTT, 0.1% bromophenol blue) and loaded onto a 12% SDS-PAGE gel. Western blots were performed as described in Lucci et al., 2020 [24], using flotilin-1 (Santa Cruz; (C7) sc-133,153, 1:1000) as an EV protein marker and anti-calnexin (SicGen: AB0041-200, 1:1000 dilution), which is depleted in small EVs (MISEV 2018; [75]). For transmission electron microscopy, EV samples were fixed in 3% glutaraldehyde in 0.1 M cacodylate buffer. 10 µls of fixed EVs were loaded onto poly-L-lysine coated copper grids and left to settle for 15 minutes. The excess liquid was wicked away with filter paper and the grids washed twice in Milli-Q water for 30 seconds, wicking away the excess water after each wash. The samples were stained with 10 µls of 2% Uranyl acetate (0.2 µm filtered) for 1 minute after which, excess stain was wicked away using filter paper and the grids was left to air dry. Grids were visualized using a Tecnai G2 T12 Biotwin transmission electron microscope (FEI) with an accelerating voltage of 100 kV and images of varying magnifications were captured using a MegaView II (Olympus) camera system.

### Axoplasm isolation from myelinated ventral and dorsal roots fibres

Sprague-Dawley male adult rats (10 months) were used for the isolation of axoplasm of motor and sensory neurons (derived from ventral and dorsal roots, respectively), as previously described [11]. Briefly, lumbar spinal roots were dissected from euthanized rats. The tissue was suspended in a modified gluconate-substituted calcium-free Cortland salt solution (132 mM Na-gluconate, 5 mM KCl, 20 mM HEPES, 10 mM glucose, 3.5 mM MgSO_4_, and 2 mM EGTA, pH 7.2) stored at 4°C. In 3–5 mm pieces, the ventral or dorsal roots were immersed in a denaturation solution (30 mM zinc acetate, 0.1 M Tricine, pH 4.8) for 10 min. Then, the roots were transferred to a 35 mm plastic culture dish containing an axon ‘pulling’ solution (40 mM aspartic acid, 38 mM Tris, 1 mM NaN_3_, and 0.02% Tween 20, pH 5.5.) in which axoplasm was translated out of its myelin sheath with a pair of micro-tweezers #5. The pulling generates a spray of axons, which was condensed into a bundle by briefly drawing the spray out of solution except for one end. Isolated axoplasm bundles were attached with the aid of eyebrow hair tools to a coverslip coated with 1% (3-aminopropyl) triethoxysilane (Sigma-Aldrich) in ethanol. The tissue that remains at the end of the bundle was removed by a scalpel and the bundles washed several times with fresh ‘pulling’ solution. The isolated axoplasm was removed from the coverslip with the aid of an eyebrow hair tool, placed in the cap of an eppendorf 1.5 mL tube in 20 ul of ‘pulling’ solution, and stored at −80°C until RNA extraction was performed.

### Axonal growth assays

For axon growth experiments, cortical primary cultures (1.75x10^5^ cells/cm^2^) in 6-well plates were transfected 24 h after plating using 5 μl/well of Lipofectamine 2000 reagent and 250 μl/well of Opti-MEM reduced-serum media (Thermo Fisher Scientific), in accordance with manufacturer’s instructions. miRCURY LNA (Locked Nucleic Acid) microRNA inhibitor (50 nM) and inhibitor control (50 nM) of miR-434-3p, miR-151-3p, miR-16-5p and miR-92a-3p (all Qiagen, Additional File 9) were used for transfections. In all cases, 1 μg pmaxFP-Green (Lonza, hereafter referred to as GFP) was co-transfected for visualization of transfected neurons. Cortical neuronal cultures were fixed in 4% paraformaldehyde (3.6% sucrose, 1x PBS, 5 mM MgCl_2_, pH 7.4; Thermo Fisher Scientific) 72 h after transfection before direct visualization. Microscope imaging was carried out using a wide-field fluorescence microscope (Axiovert 200 M, Zeiss) coupled to a CCD camera (Photometrics CoolSnap MYO) and Micro-Manager software 1.4.21. For quantification of axon length, an axon was defined as a neurite that was at least three times longer than any other neurite [24,139] and measured from the soma to the tip of the longest projection using Fiji software. Data are expressed as mean percentages of respective controls ± s.e.m (minimum of 40 axons measured per condition and experiment, in a total of 120–200 axons measured from 3 to 6 independent experiments).

### Immunofluorescence

Cortical neurons cultured on coverslips or microfluidic devices were fixed as described above using 4% (w/v) paraformaldehyde for 30 min, washed with 10 mM glycine in PBS, permeabilised in PBS/glycine-Triton (1× PBS, 10 mM Glycine, 0.2% Triton X-100; Sigma-Aldrich), blocked 1 h with 3% bovine serum albumin in PBS (BSA; Sigma-Aldrich) and further incubated with anti-acetylated tubulin (1:300; C6-11B-1, Cat no. T7451; Sigma-Aldrich), anti-Map2 (1:100; Cat. no. ab32454; Abcam) overnight followed by 1 h incubation with secondary antibodies (Alexa Fluor 488 and 568; 1:300 Molecular Probes) and mounted with Vectashield Hardset mounting media with Dapi (Vectorlabs). Imaging was conducted using a wide-field fluorescence microscope (Axiovert 200 M, Zeiss) coupled to a CCD camera (Photometrics CoolSnap MYO) and Micro-Manager software 1.4.21.

### RNA isolation

RNA was isolated by the phenol-chloroform extraction method using TRIzol® Reagent (Fisher Scientific) as described in Lucci et al. [24]. To obtain axonal RNA, cortical neurons prepared from a pooled litter of ~7-9 E16.5 embryos were cultured in microfluidic devices for 9 days *in* *vitro*. Briefly, the devices were washed twice with PBS before adding 20 µL of TRIzol to each reservoir of the axonal channel and incubating for 2 min at room temperature. A volume of 100 µL of PBS was kept in the soma channel reservoirs to prevent contamination from the neuronal somas and the efficiency of axon removal from the axon channel was monitored under the microscope. Following collection of axonal sample, the whole cell (WC) RNA fraction was obtained from the somatodendritic compartment in the same manner. Fractions from 40 to 50 devices were collected and pooled for each independent biological replicate, to a total of 3 biological replicates of axonal RNA and 3 biological replicates of WC RNA. To obtain RNA from purified EVs, 1.5 ml of TRIzol was added to the EV fraction after isolation. In both instances, total RNA was extracted following manufacturer’s instructions and resuspended in RNAse-free water (Fisher Scientific) before storage at – 80°C. For axoplasm samples, RNA isolation was performed with RNAqueous^TM^-Micro Total RNA Isolation Kit (Ambion, Invitrogen, ThermoFisher Scientific) as described previously [11]. Before the RNA isolation, the ventral or dorsal axoplasm derived from five rats were collected and pooled.

### RT-qPCR

For the miRNA expression array, two individual axonal RNA samples were probed using a custom designed miRCURY LNA Pick-&-Mix microRNA PCR Panel (Qiagen, UK #203,801). Each sample was run in duplicate reactions and UniSp3 and UniSp6 were used as inter-plate calibrators. For individual miRNA expression assays and tRNA-derived fragments’ expression assays, independent WC (n = 3), axonal (n = 3) and EV (n = 4) RNA samples were run in duplicate using individual validated and custom designed miRCURY LNA primers (Qiagen, UK). All miRCURY primer sequences are listed in Table S8. In all experiments, cDNA synthesis was conducted using the miRCURY LNA Universal cDNA synthesis kit (Qiagen, UK) according to the manufacturer’s instructions, with 10ng of total RNA. cDNA was then diluted 1:60 for all targets except miR-10a/b-5p and correspondent reference genes, for which a 1:10 dilution was used. RT-qPCR was undertaken using the miRCURY LNA SYBR Green PCR Kit (Qiagen, UK). Each sample was run in duplicate reactions and UniSp6 was used as inter-plate calibrators. For all studies, PCR amplification was carried out in the Applied Biosystems Step One Plus thermocycler, using cycling parameters recommended by Qiagen (2 min 95oC, 40 cycles:10s 95oC, 60s 56oC). Data were acquired with Applied Biosystems SDS2.3 software. Passive reference dye ROX (ThermoFisher Scientific) was included in all reactions. Ct values generated for all individual expression assays ranged from Ct 19 – Ct 33. Of note, a detailed analysis of the sncRNA-seq reads for mmu-miR-10a/b-5p demonstrated a much higher abundance of a 3p isomiR for both miRNAs in our samples (see sequences in Table S8), whereas their miRBase consensus sequences showed low expression. The RT-qPCR of mmu-miR-10a/b-5p consensus sequences (miRBase accession no. MIMAT0000648 and MIMAT0000208) showed no detectable amplification (data not shown). For this reason, we instead probed the 3p isomiRs for miR-10a/b-5p RT-qPCR as detected in our sequencing studies. Expression data were analysed by relative quantification using the comparative Ct method (2^− ΔΔCt^). miRNA and tsRNA targets’ levels were analysed as relative expression to reference (2^− ΔCt^) or to WC (2^− ΔΔCt^), using the geometric mean of miR-100-5p, miR-128-3p, miR-134-5p, miR-434-3p and let7a-5p as a reference. Selected miRNA reference genes have shown stable expression in previous in-house RT-qPCR studies on developing cortical neuronal cultures [24].

### small RNA-seq

Illumina TruSeq small RNA library and 150 bp sequencing (Illumina HiSeq) was performed by GENEWIZ Inc. (South Plainfield, NJ). The Illumina TruSeq small RNA library of extracellular vesicles (EV) samples (n = 3) cell was prepared according to the standard protocol. Due to limited input of total RNA of axonal derived samples (AX) (n = 3) (approx. up to 10 ng of total RNA), the input for Whole-Cell culture (somatodentritic compartment) samples (WC) (n = 3) was adjusted to 10 ng of total RNA. Based on Yeri et al. [140] we performed some modifications to the protocol of Illumina TruSeq small RNA library preparation: the 3′ adapter, 5′ adapter, Stop Solution, RNase Inhibitor and RT primers were diluted by 50% with RNAse free water. Additionally, during PCR amplification, the Index primer and RNA PCR primer volumes were reduced by 50%, and the volume was replaced with RNAse water. Finally, a total of 16 PCR cycles were performed for AX and WC samples. In the case of axoplasm samples, where the amounts of total RNA were less than one nanogram, the protocol was modified as described above, except that a total of 19 PCR cycles were performed. Biological replicates were barcoded, and the output reads files were separated by barcoding into different FASTQ files. The raw FASTQ data sets supporting the results of this article are available at the Sequence Read Archive repository (BioProject ID: PRJNA720703).

### small RNA-seq data analysis

The raw FASTQ files obtained from GENEWIZ were used as the input for miARma-Seq pipeline, a comprehensive tool for miRNA and mRNA analysis [141], following the user manual (v 1.7.2). Low quality reads and adapter sequences were removed with Cutadapt software [142], allowing a minimum read Phred quality of 20, and with a read length with a minimum of 18nt and a maximum of 50nt. Filtered high quality reads (Ave. 20,839,164 ± SD 5,552,376 reads, Additional File 1) were aligned to Mus musculus reference genome (GRCm38/mm10 indexed from http://bowtie-bio.sourceforge.net/bowtie2/index.shtml) using Bowtie2 [143]; with default parameters (Overall genome mapping: Ave. 83 ± SD 11, Table S1). Gene counts were performed with FeatureCounts [144] using default parameters except for strandendness considerations. For quantification of protein coding, lincRNAs, snoRNAs, snRNAs, misc_RNA, scaRNA, RNY and vtRNA we used the annotation coordinates of ensembl Mus_musculus.GRCm38.96 GTF file [145] and exon as feature-type. For miRNAs quantification the mmu miRBase v22 [146] GFF file was used. For rRNAs quantification RepeatMasker annotation (http://www.repeatmasker.org) mm10 GTF file was used. For tRNAs quantification, mouse tRNAs genome coordinates were extracted from the UCSC table browser (https://genome.ucsc.edu/cgi-bin/hgTables) UCSC GtRNAdb [147] as GTF file. For piRNAs quantification, the piRNA genomic coordinates were obtained from piRNABank [107] and converted to the GRCm38 coordinate system using the Lift Genome Annotations tool (https://genome.ucsc.edu/cgi-bin/hgLiftOver). Considering the issues previously reported for piRNA entities [108,109], we performed the alignment of the piRNA sequences against a customized ncRNA dataset (including fasta sequences of snRNAs, scaRNAs, snoRNAs, misc_RNAs, vtRNAs, RNY and miRNAs extracted from ensembl GRCm38, rRNAs from RepeatMasker and tRNAs from UCSC GtRNAdb mm10), with Bowtie2 using parameters: -N 0 -L 16 -end-to-end. Only piRNAs that did not map with our customized ncRNAs multi-fasta dataset were considered as piRNA entities for posterior analysis.

The complete raw counts of all entities were collected and considered for the global small RNA expression landscape analysis (Table S1). The complete count list was log2 transformed and Heatmap of Pearson correlation was performed using corrplot, gplots and RColorBrewer libraries; and PCA analysis was performed using rgl and plot3d libraries in RStudio (http://www.rstudio.com/). Normalization and Differential Expression Genes (DEGs) analysis were conducted using SARTools pipeline [148] in RStudio, selecting edgeR algorithm [149], upperquartile (UQ) normalization and CPM (Counts Per Million) ≥1 as cut-off (Table S2).

In order to explore and contemplate specific characteristics of the different ncRNA biotypes we performed individual analysis for miRNAs, tRNAs and sRNAs (snRNAs, snoRNAs and scaRNAs). For the miRNAs, the read counts of miRNAs which are encoded in distinct genetic loci but correspond to the identical mature miRNA sequence were collapsed, then normalization and differential expression analysis were performed using SARTools pipeline [148], selecting edgeR algorithm [149], upperquartile (UQ) normalization and CPM≥1 as cut-off (Table S3). For tRNAs, read counts of tRNAs encoded in distinct genetic loci that have the same anticodon sequence were collapsed. The collapsed tRNA counts were normalized with SARTools pipeline, selecting edgeR algorithm, upperquartile (UQ) normalization and C.P.M.≥1 as cut-off (Table S4). Furthermore, in order to annotate the different tRNA-derived fragments (tRFs), genome mapped tRNA reads were extracted using Bedtools [150] and where the input of Unitas software version 1.7.7 [102], (parameters: -insdel 2 -mismatch 2) and fractionated counts were used to compare tRFs relative abundances (Table S5). For the sRNAs (snRNAs, snoRNAs and scaRNAs), in order to count all reads mapped to sRNAs we created a customized GTF file with annotation coordinates of snRNAs, snoRNAs and scaRNAs species extracted from ensembl Mus_musculus.GRCm38.96 GTF file was used and sRNAs counts were collapsed following the RFAM [151] family type ID classification for snRNAs, snoRNAs and scaRNAs, then normalized with SARTools pipeline, selecting edgeR algorithm, upperquartile (UQ) normalization and CPM≥1 as cut-off (Table S6). The tRNAs and sRNAs feature base coverage was determined using Bedtools [150]. In all differential expression analysis, the absolute Log_2_FC ≥ 1 and False Discovery Rate (Benjamini–Hochberg-adjusted p-value) < 0.01 were considered as threshold. The same small RNA analysis strategy described for mouse samples was performed for the two axon *in* *vivo* rat samples but where Rattus norvegicus reference genome (Rnor6.0 indexed http://bowtie-bio.sourceforge.net/bowtie2/index.shtml) and annotation files from ensembl (Rnor_6.0.102), miRBase (rno_v22) and RepeatMasker (Rnor6.0) annotation were used. The motor (ARV) and sensory axons (ARD) were separately analysed (Table S7) but then presented together as average mature axon values due to globally similar results. Furthermore, in order to compare the miRNAs found with currently available miRNAs RNA-seq datasets of axons, we selected the miRNAs with CPM≥50 (approx. ≥10 reads average) found in mouse *in* *vitro* axon samples and miRNAs with at least 5 reads average counts in rat *in* *vivo* axoplasm samples.

### Pathway analysis

For pathway analysis of the 10 top-ranked miRNAs in axon and EVs we used miRPath V.3 to predict mRNA transcript targets (TargetScan v6.2, conservation score, p<0.05), and to retrieve the overrepresented Kyoto Encyclopedia of Genes and Genomes (KEGG) pathways (FDR<0.05) predicted to be targeted by each subset of miRNAs: 75 pathways in AX, 59 in EV and 96 in the ‘signature’ of 23 axonal miRNAs. For tsRNAs we applied a microRNA target prediction and pathway analysis pipeline to predict potential pathways targeted. mRNA targets of selected tsRNAs were predicted with mirTarget prediction algorithm in miRDB database (http://mirdb.org/). PANTHER (http://pantherdb.org/) was then used to retrieve those pathways potentially targeted by each set of target genes.

### Statistical analysis

All the analyses were performed in R software (version 3.6) using libraries ggcorrplot, corrplot, xlsx, gplot, heatmap.2 (clustering distance measured by Euclidean and Ward clustering algorithms) and GraphPad Prism 6 software. The probability distribution of the data set was analysed before further statistical analysis (D’Agostino-Pearson normality test). Statistical evaluation between two groups was performed using unpaired Student’s t-test. Analyses of more than two groups were carried out using two-way ANOVA with Tukey’s post-hoc analysis. Statistical significance was expressed as * p < 0.05, ** p < 0.01; two tailed p-value. The Venn diagrams were performed using jvenn [152].

## Supplementary Material

Supplemental Material

## Data Availability

The datasets supporting the conclusions of this article are available at SRA repository (ID PRJNA720703) and within the article’s supplementary information.

## Aknowledgements

The authors thank the Nanoscale and Microscale Research Centre (nmRC; University of Nottingham) for instrumentation access and Denise McLean for technical assistance with sample preparation and imaging.
