# Supplementary material for: Distinct small non-coding RNA landscape in the axons and released extracellular vesicles of developing primary cortical neurons and the axoplasm of adult nerves
Source: RNA Biol. 2021 Dec 9;18(Suppl 2):832–55. doi: 10.1080/15476286.2021.2000792 (PMC8782166; doi:10.1080/15476286.2021.2000792)
Supplement: Supplemental Material [file KRNB_A_2000792_SM1038.zip › Supplementary information/Suppl Fig S1-S6.pdf]

Suppl Fig S1

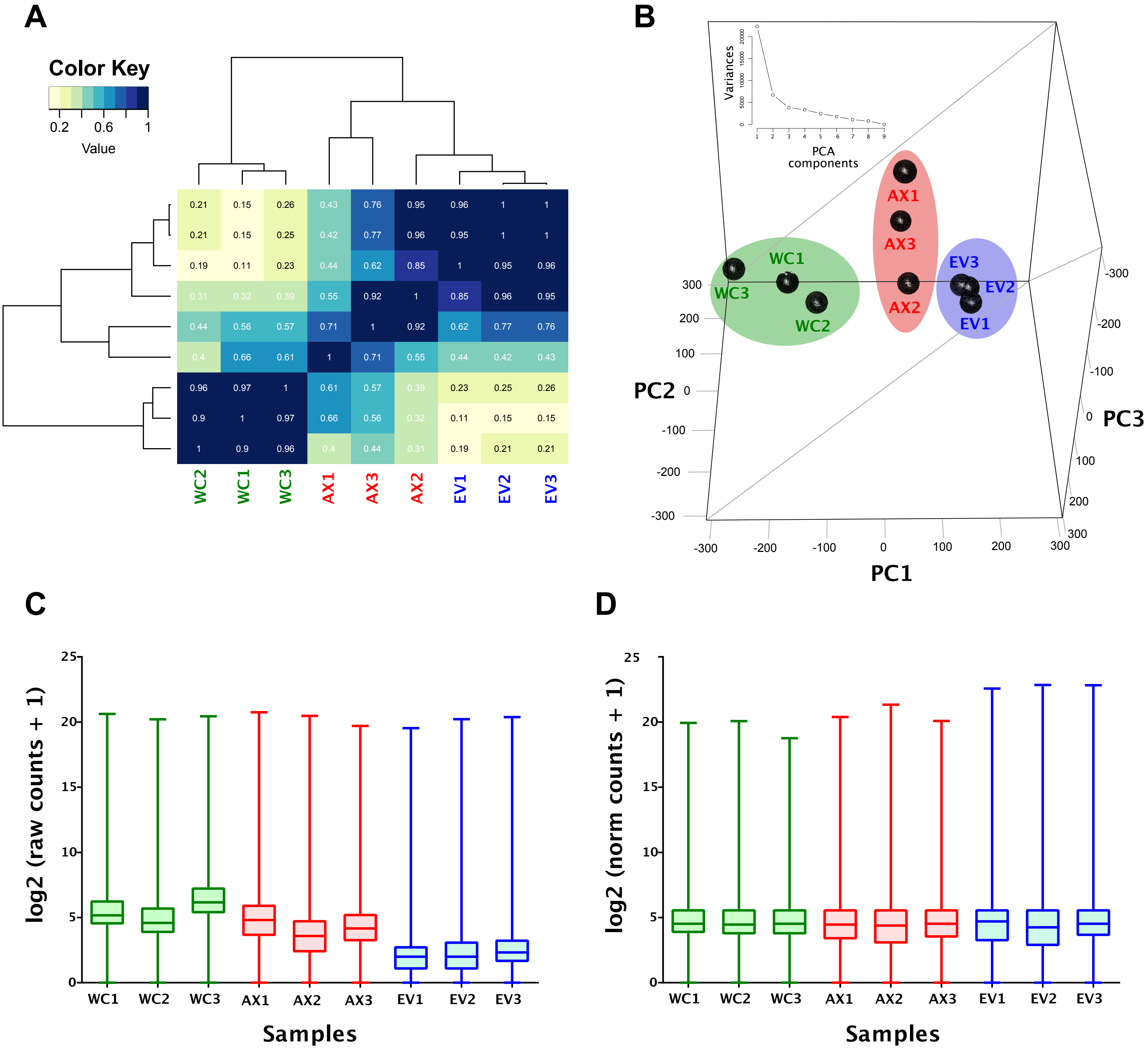

A
Color Key
0.2 0.6 1
Value
0.21 0.15 0.26 0.43 0.76 0.95 0.96 1 1
0.21 0.15 0.25 0.42 0.77 0.96 0.95 1 1
0.19 0.11 0.23 0.44 0.62 0.85 1 0.95 0.96
0.31 0.32 0.39 0.55 0.92 1 0.85 0.96 0.95
0.44 0.56 0.57 0.71 1 0.92 0.62 0.77 0.76
0.4 0.66 0.61 1 0.71 0.55 0.44 0.42 0.43
0.96 0.97 1 0.61 0.57 0.39 0.23 0.25 0.26
0.9 1 0.97 0.66 0.56 0.32 0.11 0.15 0.15
1 0.9 0.96 0.4 0.44 0.31 0.19 0.21 0.21
WC2 WC1 WC3 AX1 AX3 AX2 EV1 EV2 EV3
B
Variances
PCA components
AX1 AX3 AX2
WC1 WC3 WC2
EV3 EV2 EV1
PC1
PC2
PC3
C
log2 (raw counts + 1)
WC1 WC2 WC3 AX1 AX2 AX3 EV1 EV2 EV3
Samples
D
log2 (norm counts + 1)
WC1 WC2 WC3 AX1 AX2 AX3 EV1 EV2 EV3
Samples

Suppl Figure S2

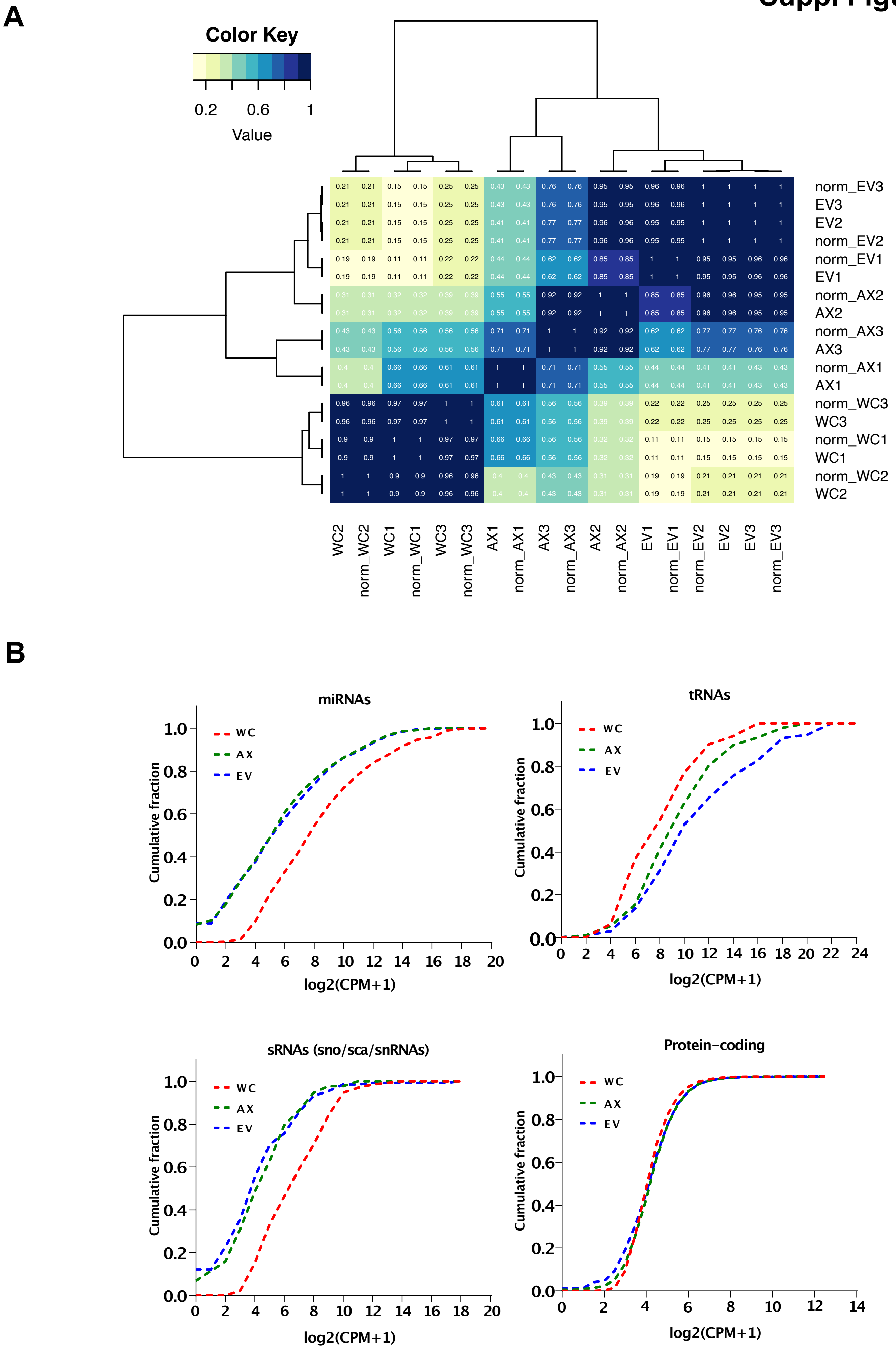

Suppl Figure S3

A

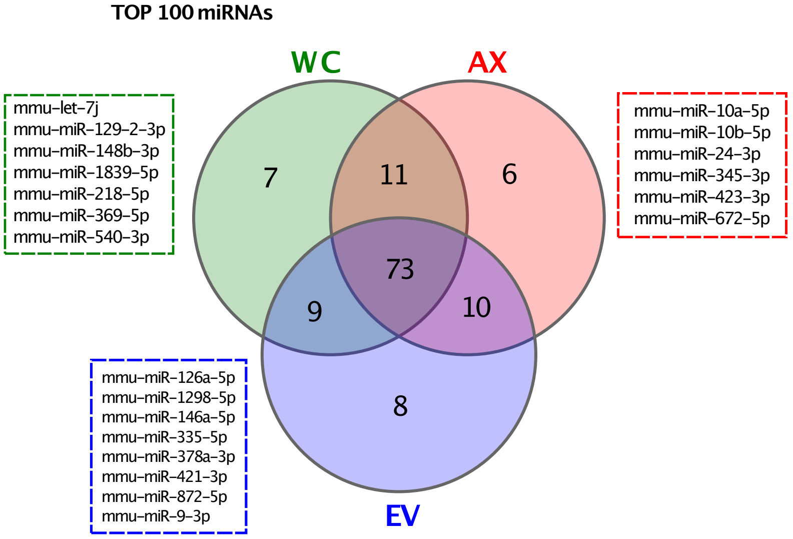

B

	mir-434-3p	miR-127-3p	miR-128-3p	miR-92a-3p	miR-9-5p	miR-486-5p	miR-191-5p
Mean	29.99	32.65	29.69	29.04	31.48	32.88	32.22
SE	0.79	0.54	1.41	0.14	0.99	0.01	1.12
	miR-26a-5p	miR-151-3p	miR-125b-3p	miR-25-3p	miR-16-5p	miR-99a-5p	miR-27b-3p
Mean	32.50	33.60	33.88	28.69	28.19	28.85	29.85
SE	0.42	0.75	0.32	0.72	0.96	0.54	0.64
	miR-93-3p	miR-30a-5p	miR-30e-3p	miR-708-5p	miR-146a-5p	miR-146b-5p	miR-26b-5p
Mean	32.84	32.90	35.08	32.85	Undert.	Undert.	Undert.
SE	0.25	0.79	1.59	1.32			

C

Selected top-ranked miRNAs in all compartments

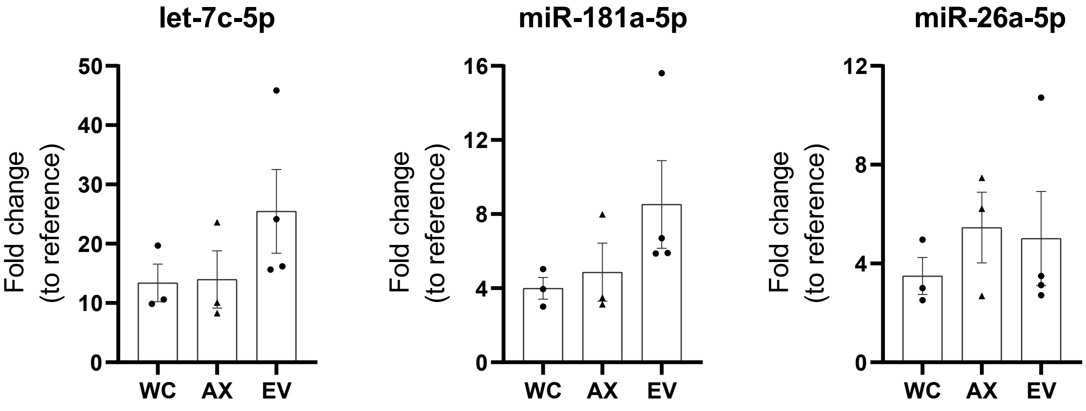

D

Selected enriched miRNAs in axon and EVs

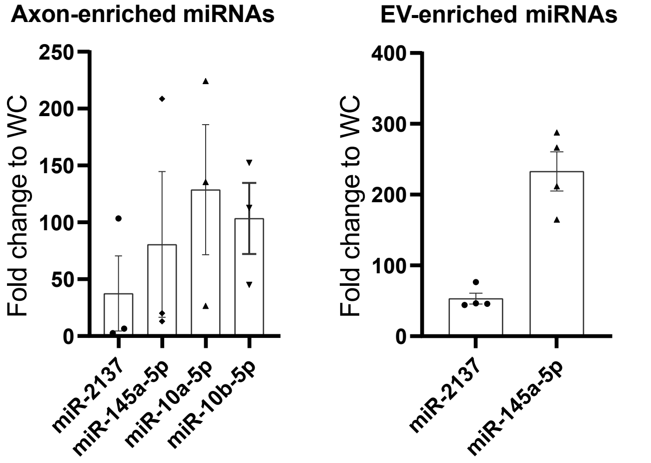

E

Selected top-ranked tsRNAs in axon and EVs

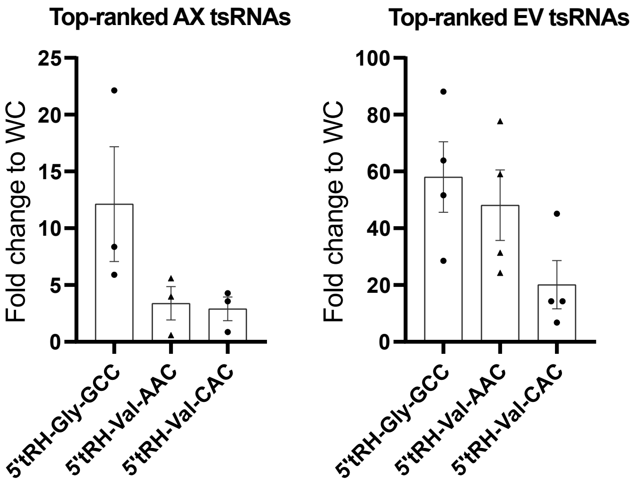

Suppl Figure S4

A

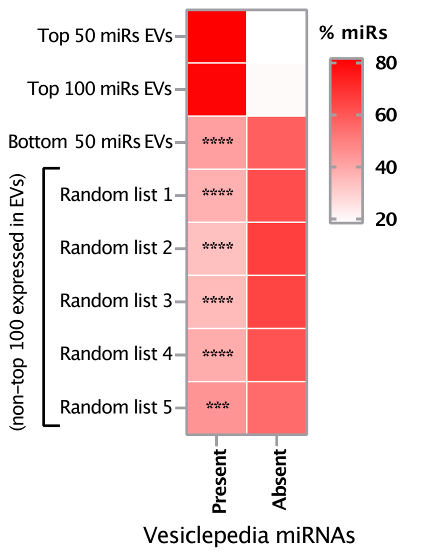

B

	Motifs								
Top 50 miRs EVs	GGAG	CCCU	UGCA	GGCU	CUGG	GCUG	GUUG	GCAG	Total # Motifs
mmu-let-7a-5p							1		1
mmu-let-7b-5p							1		1
mmu-let-7c-5p							1		1
mmu-let-7f-5p									0
mmu-let-7g-5p									0
mmu-let-7i-5p						1			1
mmu-miR-103-3p				1				1	2
mmu-miR-125a-5p		1							1
mmu-miR-125b-1-3p	1			1					2
mmu-miR-125b-5p		1							1
mmu-miR-126a-5p									0
mmu-miR-127-3p				1					1
mmu-miR-128-3p									0
mmu-miR-130a-3p			1						1
mmu-miR-136-3p									0
mmu-miR-143-3p									0
mmu-miR-149-5p				1	1				2
mmu-miR-151-3p				1					1
mmu-miR-16-5p								1	1
mmu-miR-181a-5p						1			1
mmu-miR-181b-5p						1			1
mmu-miR-181c-3p									0
mmu-miR-181c-5p							1		1
mmu-miR-186-5p				1					1
mmu-miR-191-5p						1		1	2
mmu-miR-204-5p		1							1
mmu-miR-2137	2								2
mmu-miR-22-3p						1	1		2
mmu-miR-25-3p			1						1
mmu-miR-26a-5p				1					1
mmu-miR-27b-3p				1					1
mmu-miR-298-5p	1			1		1		1	4
mmu-miR-301a-3p			1						1
mmu-miR-30a-5p					1				1
mmu-miR-30c-5p									0
mmu-miR-30d-5p					1				1
mmu-miR-30e-5p					1				1
mmu-miR-381-3p									0
mmu-miR-410-3p									0
mmu-miR-434-3p									0
mmu-miR-486a-5p						1			1
mmu-miR-486b-5p						1			1
mmu-miR-541-5p							1		1
mmu-miR-6240									0
mmu-miR-92a-3p			1						1
mmu-miR-92b-3p			1						1
mmu-miR-93-5p			1			1		1	3
mmu-miR-9-5p						1			1
mmu-miR-99a-5p									0
mmu-miR-99b-5p									0

C

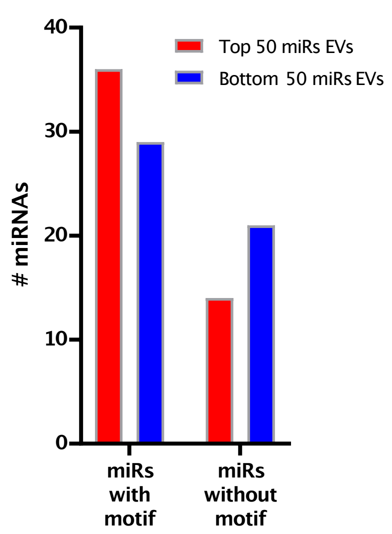

Suppl Figure S5

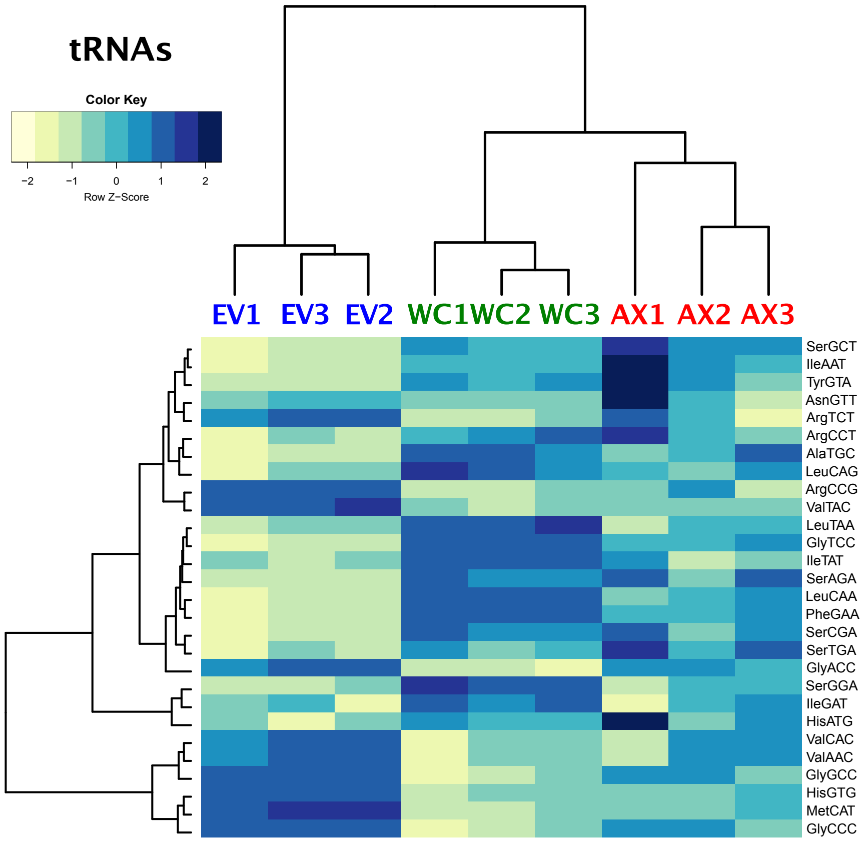

Suppl Fig S6

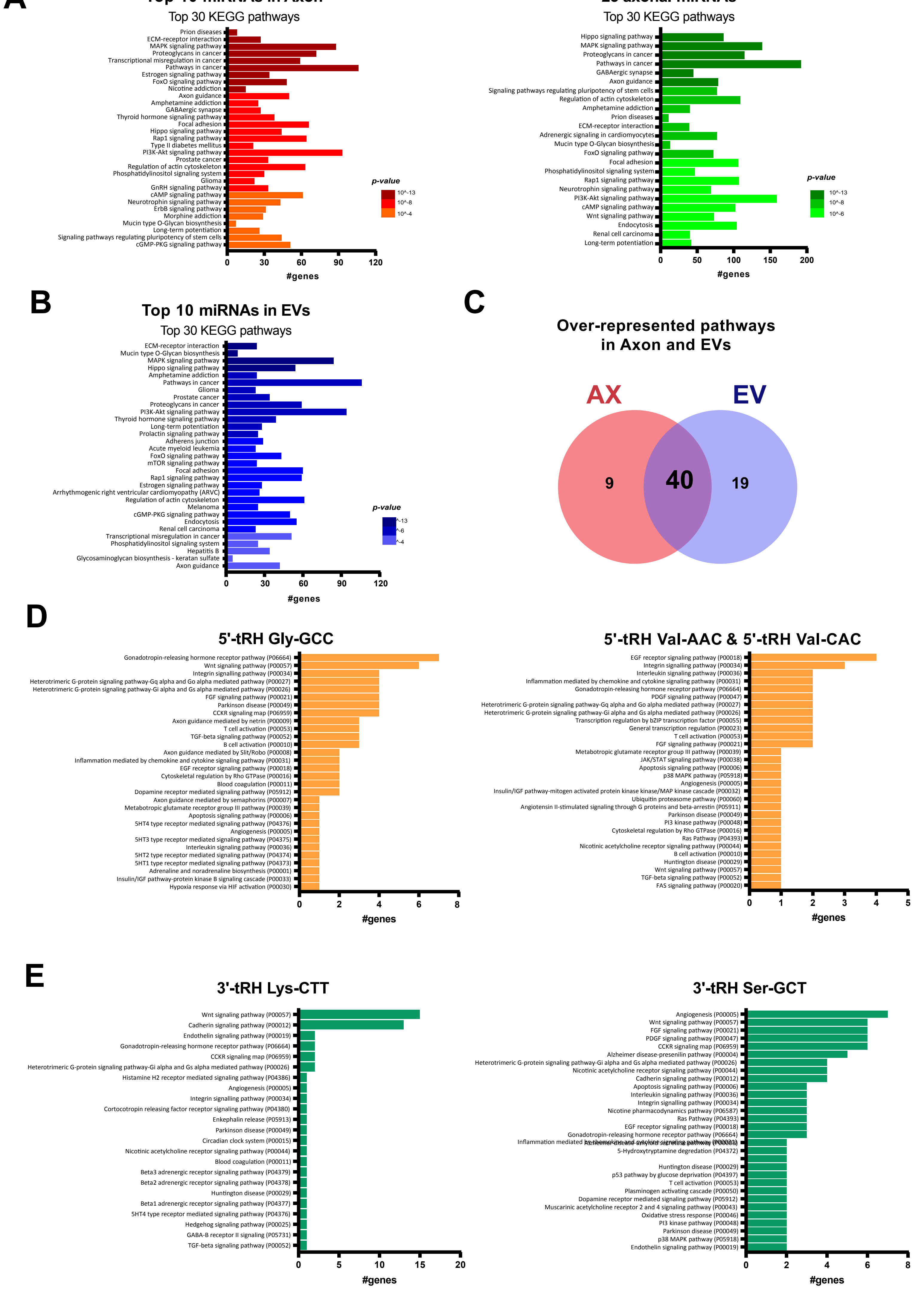
A
Top 10 miRNAs in Axon
Top 30 KEGG pathways
Prion diseases
ECM-receptor interaction
MAPK signaling pathway
Proteoglycans in cancer
Transcriptional misregulation in cancer
Pathways in cancer
Estrogen signaling pathway
FoxO signaling pathway
Nicotine addiction
Axon guidance
Amphetamine addiction
GABAergic synapse
Thyroid hormone signaling pathway
Focal adhesion
Hippo signaling pathway
Rap1 signaling pathway
Type II diabetes mellitus
PI3K-Akt signaling pathway
Prostate cancer
Regulation of actin cytoskeleton
Phosphatidylinositol signaling system
Glioma
GnRH signaling pathway
cAMP signaling pathway
Neurotrophin signaling pathway
ErbB signaling pathway
Morphine addiction
Mucin type O-Glycan biosynthesis
Long-term potentiation
Signaling pathways regulating pluripotency of stem cells
cGMP-PKG signaling pathway
0
30
60
90
120
#genes
p-value
10^-13
10^-8
10^-4
23 axonal miRNAs
Top 30 KEGG pathways
Hippo signaling pathway
MAPK signaling pathway
Proteoglycans in cancer
Pathways in cancer
GABAergic synapse
Axon guidance
Signaling pathways regulating pluripotency of stem cells
Regulation of actin cytoskeleton
Amphetamine addiction
Prion diseases
ECM-receptor interaction
Adrenergic signaling in cardiomyocytes
Mucin type O-Glycan biosynthesis
FoxO signaling pathway
Focal adhesion
Phosphatidylinositol signaling system
Rap1 signaling pathway
Neurotrophin signaling pathway
PI3K-Akt signaling pathway
cAMP signaling pathway
Wnt signaling pathway
Endocytosis
Renal cell carcinoma
Long-term potentiation
0
50
100
150
200
#genes
p-value
10^-13
10^-8
10^-6
B
Top 10 miRNAs in EVs
Top 30 KEGG pathways
ECM-receptor interaction
Mucin type O-Glycan biosynthesis
MAPK signaling pathway
Hippo signaling pathway
Amphetamine addiction
Pathways in cancer
Glioma
Prostate cancer
Proteoglycans in cancer
PI3K-Akt signaling pathway
Thyroid hormone signaling pathway
Long-term potentiation
Prolactin signaling pathway
Adherens junction
Acute myeloid leukemia
FoxO signaling pathway
mTOR signaling pathway
Focal adhesion
Rap1 signaling pathway
Estrogen signaling pathway
Arrhythmogenic right ventricular cardiomyopathy (ARVC)
Regulation of actin cytoskeleton
Melanoma
cGMP-PKG signaling pathway
Endocytosis
Renal cell carcinoma
Transcriptional misregulation in cancer
Phosphatidylinositol signaling system
Hepatitis B
Glycosaminoglycan biosynthesis - keratan sulfate
Axon guidance
0
30
60
90
120
#genes
p-value
^-13
^-6
^-4
C
Over-represented pathways
in Axon and EVs
AX
EV
9
40
19
D
5'-tRH Gly-GCC
Gonadotropin-releasing hormone receptor pathway (P06664)
Wnt signaling pathway (P00057)
Integrin signalling pathway (P00034)
Heterotrimeric G-protein signaling pathway-Gq alpha and Go alpha mediated pathway (P00027)
Heterotrimeric G-protein signaling pathway-Gi alpha and Gs alpha mediated pathway (P00026)
FGF signaling pathway (P00021)
Parkinson disease (P00049)
CCKR signaling map (P06959)
Axon guidance mediated by netrin (P00009)
T cell activation (P00053)
TGF-beta signaling pathway (P00052)
B cell activation (P00010)
Axon guidance mediated by Slit/Robo (P00008)
Inflammation mediated by chemokine and cytokine signaling pathway (P00031)
EGF receptor signaling pathway (P00018)
Cytoskeletal regulation by Rho GTPase (P00016)
Blood coagulation (P00011)
Dopamine receptor mediated signaling pathway (P05912)
Axon guidance mediated by semaphorins (P00007)
Metabotropic glutamate receptor group III pathway (P00039)
Apoptosis signaling pathway (P00006)
5HT4 type receptor mediated signaling pathway (P04376)
Angiogenesis (P00005)
5HT3 type receptor mediated signaling pathway (P04375)
Interleukin signaling pathway (P00036)
5HT2 type receptor mediated signaling pathway (P04374)
5HT1 type receptor mediated signaling pathway (P04373)
Adrenaline and noradrenaline biosynthesis (P00001)
Insulin/IGF pathway-protein kinase B signaling cascade (P00033)
Hypoxia response via HIF activation (P00030)
0
2
4
6
8
#genes
5'-tRH Val-AAC & 5'-tRH Val-CAC
EGF receptor signaling pathway (P00018)
Integrin signalling pathway (P00034)
Interleukin signaling pathway (P00036)
Inflammation mediated by chemokine and cytokine signaling pathway (P00031)
Gonadotropin-releasing hormone receptor pathway (P06664)
PDGF signaling pathway (P00047)
Heterotrimeric G-protein signaling pathway-Gq alpha and Go alpha mediated pathway (P00027)
Heterotrimeric G-protein signaling pathway-Gi alpha and Gs alpha mediated pathway (P00026)
Transcription regulation by bZIP transcription factor (P00055)
General transcription regulation (P00023)
T cell activation (P00053)
FGF signaling pathway (P00021)
Metabotropic glutamate receptor group III pathway (P00039)
JAK/STAT signaling pathway (P00038)
Apoptosis signaling pathway (P00006)
p38 MAPK pathway (P05918)
Angiogenesis (P00005)
Insulin/IGF pathway-mitogen activated protein kinase kinase/MAP kinase cascade (P00032)
Ubiquitin proteasome pathway (P00060)
Angiotensin II-stimulated signaling through G proteins and beta-arrestin (P05911)
Parkinson disease (P00049)
PI3 kinase pathway (P00048)
Cytoskeletal regulation by Rho GTPase (P00016)
Ras Pathway (P04393)
Nicotinic acetylcholine receptor signaling pathway (P00044)
B cell activation (P00010)
Huntington disease (P00029)
Wnt signaling pathway (P00057)
TGF-beta signaling pathway (P00052)
FAS signaling pathway (P00020)
0
1
2
3
4
5
#genes
E
3'-tRH Lys-CTT
Wnt signaling pathway (P00057)
Cadherin signaling pathway (P00012)
Endothelin signaling pathway (P00019)
Gonadotropin-releasing hormone receptor pathway (P06664)
CCKR signaling map (P06959)
Heterotrimeric G-protein signaling pathway-Gi alpha and Gs alpha mediated pathway (P00026)
Histamine H2 receptor mediated signaling pathway (P04386)
Angiogenesis (P00005)
Integrin signalling pathway (P00034)
Cortocotropin releasing factor receptor signaling pathway (P04380)
Enkephalin release (P05913)
Parkinson disease (P00049)
Circadian clock system (P00015)
Nicotinic acetylcholine receptor signaling pathway (P00044)
Blood coagulation (P00011)
Beta3 adrenergic receptor signaling pathway (P04379)
Beta2 adrenergic receptor signaling pathway (P04378)
Huntington disease (P00029)
Beta1 adrenergic receptor signaling pathway (P04377)
5HT4 type receptor mediated signaling pathway (P04376)
Hedgehog signaling pathway (P00025)
GABA-B receptor II signaling (P05731)
TGF-beta signaling pathway (P00052)
0
5
10
15
20
#genes
3'-tRH Ser-GCT
Angiogenesis (P00005)
Wnt signaling pathway (P00057)
FGF signaling pathway (P00021)
PDGF signaling pathway (P00047)
CCKR signaling map (P06959)
Alzheimer disease-presenilin pathway (P00004)
Heterotrimeric G-protein signaling pathway-Gi alpha and Gs alpha mediated pathway (P00026)
Nicotinic acetylcholine receptor signaling pathway (P00044)
Cadherin signaling pathway (P00012)
Apoptosis signaling pathway (P00006)
Interleukin signaling pathway (P00036)
Integrin signalling pathway (P00034)
Nicotine pharmacodynamics pathway (P06587)
Ras Pathway (P04393)
EGF receptor signaling pathway (P00018)
Gonadotropin-releasing hormone receptor pathway (P06664)
Inflammation mediated by chemokine and cytokine signaling pathway (P00031)
Alzheimer disease-amyloid secretase pathway (P00003)
5-Hydroxytryptamine degredation (P04372)
Huntington disease (P00029)
p53 pathway by glucose deprivation (P04397)
T cell activation (P00053)
Plasminogen activating cascade (P00050)
Dopamine receptor mediated signaling pathway (P05912)
Muscarinic acetylcholine receptor 2 and 4 signaling pathway (P00043)
Oxidative stress response (P00046)
PI3 kinase pathway (P00048)
Parkinson disease (P00049)
p38 MAPK pathway (P05918)
Endothelin signaling pathway (P00019)
0
2
4
6
8
#genes
