# Supplementary material for: Distinct small non-coding RNA landscape in the axons and released extracellular vesicles of developing primary cortical neurons and the axoplasm of adult nerves
Source: RNA Biol. 2021 Dec 9;18(Suppl 2):832–55. doi: 10.1080/15476286.2021.2000792 (PMC8782166; doi:10.1080/15476286.2021.2000792)
Supplement: Supplemental Material [file KRNB_A_2000792_SM1038.zip › Supplementary information/Supplementary Table 8_PCR primers and inhibitor sequences.docx]

**Supplementary Table 1** – List of RT-qPCR primers for miRNA and tRNA-derived fragments, and miRNA inhibitors used in the study

| **miRNA qPCR primers**  **(miRCURY LNA qPCR assays, Qiagen)** | **5’ Target sequence** |
| --- | --- |
| mmu-miR-26a-5p | UUCAAGUAAUCCAGGAUAGGCU |
| mmu-miR-16-5p | UAGCAGCACGUAAAUAUUGGCG |
| mmu-miR-27b-3p | UUCACAGUGGCUAAGUUCUGC |
| mmu-miR-146b-5p | UGAGAACUGAAUUCCAUAGGCU |
| mmu-miR-127-3p | UCGGAUCCGUCUGAGCUUGGCU |
| mmu-miR-191-5p | CAACGGAAUCCCAAAAGCAGCUG |
| mmu-miR-30a-5p | UGUAAACAUCCUCGACUGGAAG |
| mmu-miR-708-5p | AAGGAGCUUACAAUCUAGCUGGG |
| mmu-miR-25-3p | CAUUGCACUUGUCUCGGUCUGA |
| mmu-miR-26b-5p | UUCAAGUAAUUCAGGAUAGGU |
| mmu-miR-30e-3p | CUUUCAGUCGGAUGUUUACAGC |
| mmu-miR-9-5p | UCUUUGGUUAUCUAGCUGUAUGA |
| mmu-miR-99a-5p | AACCCGUAGAUCCGAUCUUGUG |
| mmu-miR-146a-5p | UGAGAACUGAAUUCCAUGGGUU |
| mmu-miR-125b-3p | ACGGGUUAGGCUCUUGGGAGCU |
| mmu-miR-128 | UCACAGUGAACCGGUCUCUUU |
| mmu-miR-486-5p | UCCUGUACUGAGCUGCCCCGAG |
| mmu-miR-93-3p | ACUGCUGAGCUAGCACUUCCCG |
| mmu-miR-434-3p | UUUGAACCAUCACUCGACUCCU |
| mmu-miR-151-3p | CUAGACUGAGGCUCCUUGAGG |
| mmu-miR-92a-3p | UAUUGCACUUGUCCCGGCCUG |
| miR-10a-5p  (consensus sequence in cgr) | UACCCUGUAGAUCCGAAUUUGU |
| ccr-miR-10b-5p  (consensus sequence in ccr) | UACCCUGUAGAACCGAAUUUGU |
| mmu-miR-134-5p | UGUGACUGGUUGACCAGAGGGG |
| mmu-let-7a-5p | UGAGGUAGUAGGUUGUAUAGUU |
| mmu-let-7c-5p | UGAGGUAGUAGGUUGUAUGGUU |
| mmu-miR-100-5p | AACCCGUAGAUCCGAACUUGUG |
| mmu-miR-181a-5p | AACAUUCAACGCUGUCGGUGAGU |
| mmu-miR-145a-5p | GUCCAGUUUUCCCAGGAAUCCCU |
| mmu-miR-2137 | GCCGGCGGGAGCCCCAGGGAG |

| **tRNA-derived fragments**  **qPCR primers**  **(miRCURY LNA qPCR assays, Qiagen)** | **5’ Target sequence**  **(custom design)** |
| --- | --- |
| 5’-tRH Gly-GCC | GCATTGGTGGTTCAGTGGTAGAATTCTCGCC |
| 5’-tRH Val-CAC | GTTTCCGTAGTGTAGTGGTTATCACGCTCGCCT |
| 5’-tRH Val-AAC | GTTTCCGTAGTGTAGTGGTTATCACGTTCGCCT |
| **miRNA functional assays** | **Oligo sequence**  **(miRCURY LNA inhibitor, Qiagen)** |
| miRCURY miRNA  Inhibitor Control A | TAACACGTCTATACGCCCA |
| mmu-miR-92a-3p | AGGCCGGGACAAGTGCAAT |
| mmu-miR-151-3p | CTCAAGGAGCCTCAGTCTA |
| mmu-miR-434-3p | GGAGTCGAGTGATGGTTCAA |
| mmu-miR-16-5p | GCCAATATTTACGTGCTGCT |
