# Supplementary material for: Distinct small non-coding RNA landscape in the axons and released extracellular vesicles of developing primary cortical neurons and the axoplasm of adult nerves
Source: RNA Biol. 2021 Dec 9;18(Suppl 2):832–55. doi: 10.1080/15476286.2021.2000792 (PMC8782166; doi:10.1080/15476286.2021.2000792)
Supplement: Supplemental Material [file KRNB_A_2000792_SM1038.zip › Supplementary information/Suppl_Fig_Legends_Mesquita_Ribeiro_et_al_2021.docx]

**Suppl Fig S1**. Clustering and normalization of the small RNAseq samples. (A). Heatmap of raw read counts Pearson’s correlations coefficients of the samples (two-way hierarchical clustering distance measured by Euclidean and Ward clustering algorithms) shows high correlation within biological replicates of each neuronal compartment, with WC and EV being the most distant (17,867 total features). (B) 3D principal component analysis (PCA) plot of the log2 raw reads counts of the samples indicates the clustering of the independent biological replicates analysed for each neuronal compartment, an indication of good reproducibility amongst independent samples. Variances PCA components plot (17,867 total features, inset) shows that the 3 components analysed represent the majority of the variance in the data. Box plot distribution of the samples before (C) and after (D) upper quartile (UQ) edgeR normalization and CPM ≥ 1 (count per million) filter (4953 total features). Whole Cell (WC), Axon (AX) and Extracellular Vesicles (EV).

**Supplementary Fig S2**. (A) Heatmap of Pearson correlations coefficients of the different samples for raw reads counts and normalized features using a CPM ≥ 1 (count per million) filter (4953 features) (two-way hierarchical clustering distance measured by Euclidean and Ward clustering algorithms). (B) Cumulative distribution of Log2(CPM+1) values of miRNAs, tRNAs, sRNAs and protein coding genes in the different compartments. Raw reads samples: Whole Cell (WC), Axon (AX) and Extracellular Vesicles (EV); or normalized upperquartile (UQ) edgeR Whole Cell normalized (WC_norm), Axon normalized (AX_norm) and Extracellular Vesicles normalized (EV_norm).

**Supplementary Fig S3**. miRNAs in different compartments and validation of sncRNAs by RT-qPCR. (A) Venn diagram depicting the number of common miRNAs among the more abundant top 100 in the WC, AX and EV compartments (features 563). Of those, 73 miRNAs overlap in all three compartments. Dotted boxes indicate those miRNAs from each of the Top 100 rankings that are exclusive to the specific sample type. (B) miRNA-qPCR panel probed with total RNA from cortical axons, n=2 (C) RT-qPCR of selected top expressed miRNAs in all compartments (WC, AX and EV). Data presented as fold change to reference (2^−ΔCt^). (D) RT-qPCR of selected miRNAs found to be enriched in the axon and EV of cortical neurons by snRNA-seq. Data presented as fold change to WC after normalisation to reference (2^−ΔΔCt^). (E) RT-qPCR of selected most abundant tsRNA sequences in axon and EV samples of cortical neurons. The specific RT-qPCR assays were performed in three independent samples from WC and AX, and four for EVs. Data presented as fold change to WC after normalisation to reference (2^−ΔΔCt^). For all datasets, the geometric mean of miR-100-5p, miR-128-3p, miR-134-5p, miR-434-5p used as reference (Lucci et al 2020).

**Supplementary Fig S4**. Analysis of EV sorting motifs in miRNAs. (A) Heatmap of the percentage of miRNAs present or absent in the list of 277 *Mus musculus* miRNAs reported as EV miRNAs in Vesiclepedia (http://microvesicles.org/). Lists of evaluated miRNAs include: the top 50 and top 100 miRNAs expressed in EVs, the bottom 50 miRNAs expressed in EVs (minimum CPM≥50 in WC samples) and 5 lists of 50 randomly selected miRNAs that are outside the top 100 EV miRNAs. Fisher’s exact test was applied to the comparisons of top 50 vs bottom 50 miRNAs expressed in EVs and each of the 5 random lists (**** p-value < 0.0001, *** p-value < 0.001). (B) Number of EV sorting motifs found in the mature sequences of the top 50 miRNAs expressed in EVs. (C) Contingency plot for the total number of miRNAs with or without EV sorting motifs in the top and bottom 50 miRNAs expressed in EVs (minimum CPM≥50 in WC samples).

**Supplementary Fig S5**. Heatmap of the differential expression analysis of the parental tRNAs, precursors of tRNA-derived RNAs (28 DEGs, FDR ≤ 0.01 and absolute log2(FC) ≥ 1) for all WC, AX and EV samples. Expression is shown as Z-score of log2 normalized counts (two-way hierarchical clustering distance measured by Euclidean and Ward clustering algorithms).

**Supplementary Fig S6**. Pathway analysis of top miRNAs and tRNA-derived fragments in axon and extracellular vesicle (EV) fractions. **A.** Top 30 KEGG pathways predicted to be targeted by the 10 most abundant miRNAs in the axon (AX) of cortical neurons (left; total of 75 significantly overrepresented pathways) and by the “23 axon miRNA signature” common to all axon datasets analysed in this study (right; total of 96 overrepresented pathways). **B.** Top 30 KEGG pathways predicted to be targeted by the 10 most abundant miRNAs in neuron-derived EV samples (total of 59 overrepresented pathways). Target gene predictions of top 10 most expressed miRNAs were obtained with TargetScan algorithm and mirPath v.1.3 was used to retrieve KEGG pathways overrepresented in AX and EV miRNA target lists (FDR<0.05). Data presented as #gene hits per pathway. Pathways are presented in ascending order of p-value (colour coded, see legend). **C.** Venn diagram showing the high overlap between pathways targeted by the 10 top-ranked miRNAs expressed in AX and EV samples. **D.** Pathway analysis of the most abundant tRNA-derived fragments in axon and EV fractions (5’-tRH-Gly-GCC, 5’tRH-Val-AAC and 5’tRH-ValCAC) fractions, and in the **E.** axoplasm of peripheral nerves (3’-tRH-Lys-CTT, 3’-tRH-Ser-GCT). Graphs show the top 30 pathways predicted to be targeted by the individual tRNA-derived fragments. Target predictions were performed using the mirTarget algorithm and assume miRNA-like binding to target mRNA. PANTHER Classification System (http://www.pantherdb.org/) was used to predict pathways that could be targeted by each tRNA-derived fragment. As the sequences of 5’tRH-Val-AAC and 5’tRH-ValCAC only differ at the 3’ end, mRNA target predictions, and thus pathway analysis, overlap for both tRNA halves. Data presented and ordered by #gene hits per pathway.
